# Nitrogen Fixation Mutants of the Actinobacterium *Frankia Casuarinae* CcI3

**DOI:** 10.1264/jsme2.ME17099

**Published:** 2017-11-18

**Authors:** Ken-ichi Kucho, Daiki Tamari, Shintaro Matsuyama, Takeshi Nabekura, Louis S. Tisa

**Affiliations:** 1 Graduate School of Science and Engineering, Kagoshima University 1–21–35 Korimoto, Kagoshima 890–0065 Japan; 2 Faculty of Science, Kagoshima University 1–21–35 Korimoto, Kagoshima 890–0065 Japan; 3 Department of Molecular, Cellular, and Biomedical Sciences, University of New Hampshire 289 Rudman Hall, 46 College Road, Durham, NH 03824–2617 USA

**Keywords:** genetics, genome, multicellular bacteria, *nif* genes, vesicle

## Abstract

*Frankia* is a representative genus of nitrogen-fixing (N_2_-fixing) actinobacteria; however, the molecular mechanisms underlying various phenomena such as the differentiation of a N_2_ fixation-specific structure (vesicle) and the regulation of N_2_ fixation (*nif*) genes, have yet to be elucidated in detail. In the present study, we screened hyphal fragments of *Frankia casuarinae* that were mutagenized by 1-methyl-3-nitro-1-nitrosoguanidine or gamma rays, and isolated 49 candidate N_2_ fixation mutants. Twelve of these mutants were selected for further study, and their abilities to grow in NH_3_-deficient (N-) liquid media and their rates of acetylene reduction activities were evaluated. Eleven mutant strains were confirmed to lack the ability to fix N_2_. Five mutant strains formed significantly reduced numbers of vesicles, while some failed to form large mature vesicles. These vesicle mutants also exhibited an aberrant hyphal morphology, suggesting a relationship between vesicle differentiation and hyphal branching. Ten mutants showed significant reductions in the expression of *nifE*, *nifH*, *and nifV* genes under N- conditions. The genome sequencing of eight mutants identified 20 to 400 mutations. Although mutant strains N3H4 and N6F4 shared a large number of mutations (108), most were unique to each strain. Mutant strain N7C9 had 3 mutations in the *nifD* and *nifH* genes that may result in the inability to fix N_2_. The other mutant strains did not have any mutations in any known N_2_ fixation-related genes, indicating that they are novel N_2_ fixation mutants.

Nitrogen is an essential element for all living organisms. Nitrogen fixation (N_2_ fixation) is a reaction that reduces dinitrogen (N_2_) to ammonium (NH_3_), which is then assimilated by most organisms such as bacteria, fungi, and plants; therefore, it plays an important role in the supply of a nitrogen source to ecology. Only prokaryotes are known to be N_2_-fixing organisms. Although N_2_-fixing organisms are found sporadically, they cover a wide variety of taxonomic groups from eubacteria to archaea (28). N_2_-fixing bacteria are dominantly found in α-*Proteobacteria*, γ-*Proteobacteria*, and *Cyanobacteria*, including intensively studied bacteria such as rhizobia, *Klebsiella*, *Azotobacter*, and *Anabaena*.

N_2_ fixation is catalyzed by the complex metalloenzyme nitrogenase, which is composed of dinitrogenase (NifDK) and dinitrogenase reductase (NifH) (8). The amino acid sequences of these enzymes are conserved among all N_2_-fixing species, indicative of a single evolutionary origin. In addition to the structural genes (*nifDKH*) of the nitrogenase complex, genes involved in metallocofactor synthesis are required to form an active enzyme and they are also widely conserved (9).

The transcription of N_2_-fixing genes (*nif* genes) is activated in the absence of fixed nitrogen, such as NH_3_. In *Proteobacteria*, NifA and RpoN play a key role in NH_3_-responsive regulation (8). The promoter of *nif* operons consists of consensus −12 and −24 sequences and RpoN, which encodes the RNA polymerase sigma factor σ^54^, binds to them. σ^54^ has no homology with other sigma factors, such as the σ^70^ family, and RNA polymerase associated with σ^54^ cannot form an open promoter complex (6). NifA interacts with σ^54^ and promotes the formation of an open promoter complex to initiate transcription (18).

*Frankia* spp. are representatives of N_2_-fixing actinobacteria and have several unique properties. *Frankia* fixes N_2_ not only under free-living conditions, but also in symbiosis with non-legume plant species (>200) belonging to 8 families called actinorhizal plants (12, 15). Under N_2_-fixing conditions (nitrogen-limited environments or in symbiotic nodules, except for *Casuarina* and *Allocasuarina*), *Frankia* develops vesicles, which are spherical multicellular structures devoted to N_2_ fixation (12). These vesicles are surrounded by several layers of hopanoid lipid envelopes that function as a barrier for oxygen (5). Nitrogenase, which is an oxygen-labile enzyme, is exclusively expressed in these vesicles (17). Thus, *Frankia* even fixes N_2_ under an atmospheric level of oxygen. Cellular structures that are specific for N_2_ fixation are rarely found in nature. Besides vesicles, the only other known example is the heterocyst, which is developed by multicellular cyanobacteria such as *Anabaena* and *Nostoc* (1). However, the heterocyst differs morphologically from *Frankia* vesicles and its outer layers, which block oxygen penetration, are composed of glycolipids and polysaccharides rather than hopanoids (3, 7). These findings indicate that the evolutionary origins of the two structures differ and *Frankia* independently acquired the ability to develop this N_2_-fixing structure.

*Frankia* genomes (20) do not contain many of the key regulators of *nif* genes found in *Proteobacteria* (including *nifA*, *nifL*, and *rpoN*) (8), indicating that the mechanisms responsible for NH_3_-responsive transcriptional regulation differ from these well-studied bacteria. In *Anabaena*, heterocyst differentiation is governed by regulator genes such as *ntcA* and *hetR* (1), whereas *Frankia* genomes lack homologs of these genes, again indicating different origins for the vesicle and heterocyst. Therefore, we failed to identify *Frankia* genes involved in *nif* gene regulation and vesicle differentiation using a homolog-based strategy.

In an effort to identify these *Frankia*-specific genes with a forward genetic approach, we developed a method to isolate loss-of-function mutants using *Frankia* hyphae (13). In this “fragmentation and filtration” (FF) method, we grew mutagenized hyphae at their tips, fragmented them by ultrasonic waves, and purified short fragments by filtration with a 5-μm pore. Most of the short hyphae fragments consisted of clonal mutant cells and we successfully obtained several loss-of-function mutants (13). In the present study, we applied the FF method to the isolation of N_2_-fixing mutants of *Frankia*.

## Materials and Methods

### Bacterial strain

*Frankia casuarinae* (strain CcI3) (21), which is a symbiont of *Casuarina* and *Allocasuarina* plant species (29), was used as the parental wild-type (WT) strain. Cultures were grown and maintained in liquid BAP-T medium, which contained NH_3_ as the main nitrogen source (14).

### Mutagenesis by 1-methyl-3-nitro-1-nitrosoguanidine (NTG)

*F. casuarinae* was grown in liquid BAP-T medium for approximately 7 d. Hyphae were collected by centrifugation (2,500×*g*, 20°C, 10 min) from a 14-mL culture and resuspended in 10 mL TM buffer (50 mM Tris-HCl [pH 8.0] and 50 mM maleate). NTG (Tokyo Chemical Industry, Tokyo, Japan) was added to the cell suspension at a final concentration of 1 or 2 mg mL^−1^. Cell suspensions were incubated at room temperature for the time indicated in Table 1 with gentle agitation. Hyphae were collected by centrifugation and resuspended in sterilized water to remove NTG. The washing procedure was performed twice and hyphae were then suspended in 1.1 mL BAP-T medium. Before and after mutagenesis, samples of the cell suspension were plated on solid CB media (4) to assess survival rate. The cell suspension (0.1 mL) was inoculated into 10 mL CB liquid medium and incubated at 28°C for a few weeks.

### Mutagenesis by gamma rays (GR)

*F. casuarinae* was grown in liquid CB medium for approximately 7 d. Hyphae were collected by centrifugation (2,500×*g*, 20°C, 10 min) from a 14-mL culture and resuspended in 3 mL CB medium. Hyphae were homogenized by forced passages through a 21G needle (TERUMO, Tokyo, Japan). Fragmented hyphae were irradiated with GR (^60^Co) for 8 h (772 Gy) or 12 h (1158 Gy). After irradiation, hyphae were collected by centrifugation and resuspended in 1.1 mL BAP-T medium. Before and after mutagenesis, samples of the cell suspension were plated on solid CB media to assess survival rates. The cell suspension (0.1 mL) was inoculated into 10 mL CB liquid medium and the cultures were incubated at 28°C for a few weeks.

### Preparation of colonies consisting of clonal mutant cells

We followed the FF method as described previously (13). Mutagenized hyphae cultured in CB medium (2 mL) for a few weeks were transferred to a 15-mL centrifuge tube (Greiner, Tokyo, Japan) and fragmented using the SoniMix ultrasonic homogenizer UX-050 (Mitsui Electric, Chiba, Japan) with an output power setting of 50% for 10 s. Five hundred microliters of homogenized hyphae were transferred to Ultrafree centrifugal filter units (5-μm pore; Millipore, Billerica, MA, USA) and centrifuged at 12,000×*g* for 1 min. The filtrate was spread onto solid CB medium and incubated at 28°C for one month to generate colonies.

### Screening of N_2_ fixation mutants

The first screening of N_2_-fixing mutants was performed using a previously described method (13). Briefly, eight-strip PCR tubes were filled with glass beads (As One, Osaka, Japan) and 100 μL BAP medium (2) without NH_3_ (BAP-). A colony was picked up by a sterilized toothpick and placed in each tube, and tubes were vigorously agitated by a vortex (Scientific Industries, Bohemia, NY, USA) for 5 min to homogenize the colony clump. Homogenates (3 μL) were spotted onto solid CB minimal medium containing NH_4_Cl (CBmin, CB medium deprived of Proteose Peptone No. 3) and one lacking NH_4_Cl (CBminN-). Plates were incubated at 28°C for 1 month or longer.

Strains that did not grow on CBminN- were subjected to second screening. Cells of the mutant candidates were taken from the corresponding CBmin plates, transferred to 1.5-mL microtubes filled with 100 μL CB liquid medium, and homogenized with a pestle (Scientific Specialties, Lodi, CA, USA). Homogenates were inoculated into 1 mL CB liquid medium in 24-well microtiter plates (TPP, Trasadingen, Switzerland) and incubated at 28°C for 2 weeks. Hyphae were collected from 0.5 mL culture medium by centrifugation (2,500×*g*, 20°C, 10 min), washed with sterilized water twice, and resuspended in 2 mL sterilized water. The cell suspension was subjected to the FF method as described above. The filtrate was plated on CBmin and CBminN- media and grown at 28°C for 4 to 5 weeks. Strains that did not grow on CBminN-, but grew on CBmin were subjected to third screening. In each strain, we picked up to 8 single colonies from CBmin plates of the second screening. Colonies were homogenized with glass beads in eight-strip PCR tubes as described above and homogenates (3 μL) were spotted onto CBmin and CBminN- plates. A colony that showed the most prominent difference in growth between the two media was selected and cultivated for further study.

### Growth analysis in liquid media

**Fig. 1:**
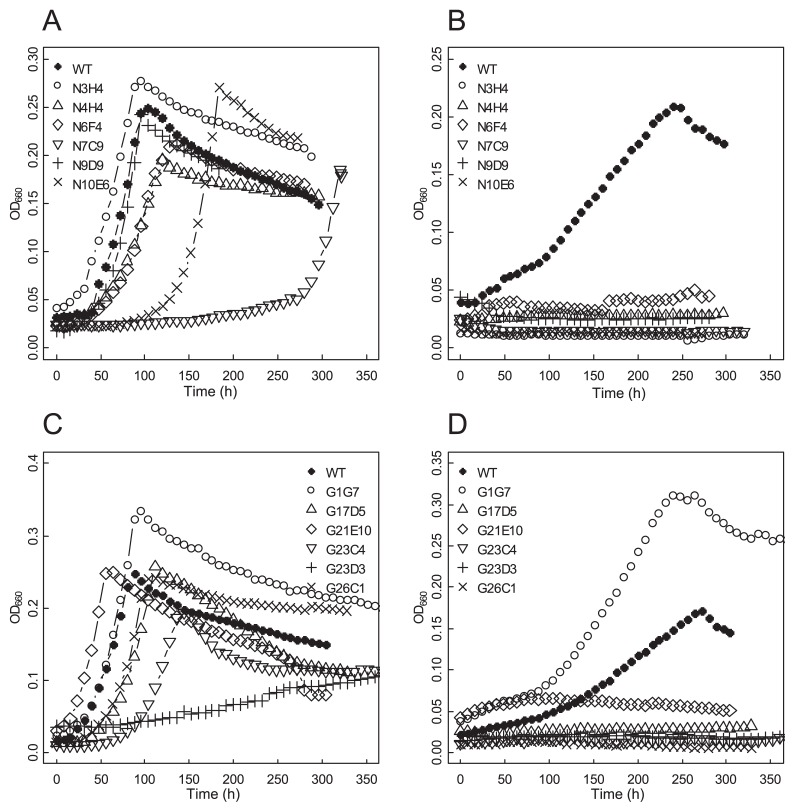
Growth curve of mutant strains. Mutant strains generated by NTG (A and B) or GR (C and D) mutagenesis were cultivated in N+ (A and C) or N- (B and D) liquid media.

*Frankia* cells precultured in BAP-T medium to the mid-log phase were collected by centrifugation (2,500×*g*, 20°C, 10 min) and washed with sterilized water twice. Hyphae were homogenized by forced passage through a 21G needle and inoculated into 150 mL BAP-T medium with NH_3_ and 150 mL BAP-T deprived of NH_3_ (BAP-TN-) in 250-mL glass media bottles (Thermo Fisher Scientific, Yokohama, Japan) at an initial concentration of OD_660_=0.02. We cultured the cells with stirring at 28°C and continuously monitored cell density using a self-made device constructed with the analog fiber sensor FX-11A (Panasonic Industrial Device, Aichi, Japan) and multichannel recorder MCR-4V (T and D, Nagano, Japan). Data were collected every 30 min and plotted every 8 h (Fig. 1).

### Acetylene reduction activity (ARA)

*Frankia* cells precultured in 150 mL BAP-T medium to the mid-to late-log phase for approximately 7 d were collected by centrifugation (2,500×*g*, 20°C, 10 min) and washed with sterilized water twice. Hyphae were transferred to 150 mL BAP-TN- in 250-mL glass media bottles (Thermo Fisher Scientific) and incubated at 28°C with stirring. After 5 to 9 d, 5 mL of the culture was transferred to a 7-mL vacutainer (BD Biosciences, Sparks, MD, USA) and 5% (v/v) acetylene was injected. After a 4-h incubation at 28°C, 1 mL of the gas phase was analyzed by gas chromatography (GC8-AIF; Shimadzu, Kyoto, Japan) to quantify the amount of ethylene generated. Total protein was extracted using NaOH (26) and concentrations were measured with the Bradford assay (Protein Assay; Bio-Rad, Hercules, CA, USA) using bovine serum albumin as the standard. Ethylene generated in 1 h by cells equivalent to 1 mg of total protein was represented as ARA.

### Observation and counting of vesicles

*Frankia* cells acclimated to NH_3_-deficient (N-) conditions were prepared as described for ARA measurements. A sample of the culture was taken 7 d after being transferred to BAP-TN- medium, and vesicles and hyphae were observed by a differential interference contrast (DIC) microscope (Eclipse TE2000-U; Nikon, Tokyo, Japan) and phase-contrast microscope (IX-70; Olympus, Tokyo, Japan). The number of vesicles was counted according to the method described previously (27). Briefly, we placed 2 mL of the culture in a 15-mL centrifuge tube (Greiner) and fragmented hyphae using the SoniMix ultrasonic homogenizer UX-050 (Mitsui Electric) with an output power setting of 38% for ~30 s. We applied the cell suspension to the interspace between a microscope slide and coverslip, the height of which was set to 0.01 mm using carbon steel tape (MonotaRO, Amagasaki, Japan). The prepared slide was observed using the DIC microscope and 5 to 9 pictures (area=0.345 mm^2^ and volume=0.00345 mm^3^) were taken. The number of vesicles in each picture were counted and normalized by the protein content of the culture. Total protein concentrations were measured with the procedure described above.

### Quantitative reverse-transcription (qRT)-PCR

*Frankia* cells were acclimated to N- conditions as described above for ARA measurements. After 4 d in BAP-TN- medium, cells were collected by centrifugation (2,500×*g*, 20°C, 10 min) and total RNA was purified by the cetyltrimethylammonium bromide (CTAB) method as described previously (14). Contaminating DNA was removed by a treatment with the TURBO DNA-free kit (Thermo Fisher Scientific). cDNA was synthesized using a PrimeScript RT reagent Kit (Perfect Real Time; Takara Bio, Ohtsu, Japan). Briefly, a 20-μL reaction mixture contained 2 μg total RNA and 2 pmol gene-specific reverse primers (Table S1). The reverse transcription reaction was incubated at 42°C for 15 min, followed by an incubation at 50°C for 15 min. Real-time PCR was performed using the TaqMan Gene Expression Master Mix (Applied Biosystems, CA, USA) and StepOnePlus Real Time PCR System (Applied Biosystems). The reaction mixture contained 2 pmol gene-specific forward and reverse primers (Table S1), 1.5 pmol TaqMan probe (Table S1), and cDNA derived from 0.3 ng (16S rRNA, internal standard) or 100 ng (*nifE*, *nifH*, and *nifV*) total RNA in a total volume of 10 μL.

### Genome analysis

Genomic DNA was purified by the CTAB method as described previously (14). Regarding strains N3H4, N4H4, N6F4, N7C9, N9D9, and N10E6, standard Illumina shot-gun libraries were constructed and sequenced using the Illumina HiSeq2500 platform, which was performed at the Hubbard Center for Genome Studies (University of New Hampshire, Durham, NH, USA). The 250-bp reads were trimmed, mapped, and assembled to the *F. casuarinae* (CcI3) genome (NC00777) using CLC Genomics workbench version 8.5. The resequencing parameter was used and the assembled mutant genomes were analyzed under a ploidy variant detection algorithm with a variant probability setting at 90%. Regarding strains G21E10 and G23C4, sequencing (Illumina HiSeq4000 platform) and variant detection were performed by BGI genomics (Kobe, Japan).

The following known N_2_ fixation-related genes were checked for mutations: nitrogenase (*nif*) genes (Francci3_4472 to Francci3_4489), hydrogenase (*hup*) genes (Francci3_1937 to Francci3_1948 and Francci3_1069 to Francci3_1079), sulfur iron co-factor (*suf*) genes (Francci3_1660 to Francci3_1666), and hopanoid synthesis genes (Francci3_0818 to Francci3_0826, Francci3_1326, Francci3_3573, Francci3_3575, Francci3_3958, Francci3_4188, Francci3_4253 and Francci3_4254) (2).

## Results

### Mutant screening

We screened our libraries for potential mutants that showed reduced growth on N- solid medium, but grew well on NH_3_-replete (N+) solid medium (Table 1). Among 3,534 NTG-and 3,248 GR-mutagenized colonies, 30 and 19 mutant strains were isolated, respectively, and were considered to be candidates for N_2_ fixation-defective mutants. The occurrence of these mutant strains was slightly more frequent with NTG mutagenesis (0.8%) than GR (0.6%). This rate of occurrence negatively correlated with the survival rate. Six NTG-mutagenized strains (N3H4, N4H4, N6F4, N7C9, N9D9, and N10E6) and 6 GR-mutagenized strains (G1G7, G17D5, G21E10, G23C4, G23D3, and G26C1) were selected for further detailed characterization (Table 1).

### Growth in liquid media and ARA

In N+ liquid medium, 10 of the mutant strains grew at a similar rate to the parental WT strain. Mutant strains N7C9 and G23D3 showed a slower growth rate than the parental WT (Figs. 1A and C). In N- liquid medium, 11 mutant strains showed no increase in growth for more than 10 d (Figs. 1B and D). Under these conditions, WT cells grew to a similar density as that under N+ conditions. One GR-mutagenized strain (G1G7) grew vigorously under N- conditions and was considered to be a false-positive selection from our original screening.

ARA was measured to evaluate N_2_ fixation activity. Between 5 and 9 d after their transfer to N- conditions, WT and mutant G1G7 apparently showed ARA (Table 2). In contrast, the 11 other mutant strains (N3H4, N4H4, N6F4, N7C9, N9D9, N10E6, G17D5, G21E10, G23C4, G23D3, and G26C1) did not show a significant peak in ethylene, demonstrating that they lacked the ability to fix N_2_ (Table 2).

### Vesicle formation

**Fig. 2:**
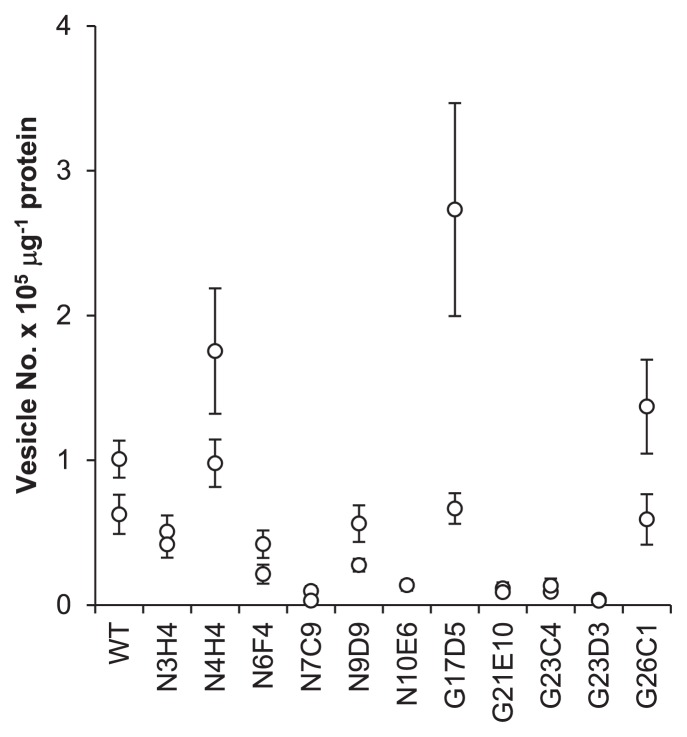
Number of vesicles. Data from two biological replicates are shown for each strain. The bar represents the standard deviation of 5 to 9 microscopic images (see Materials and Methods).

**Fig. 3:**
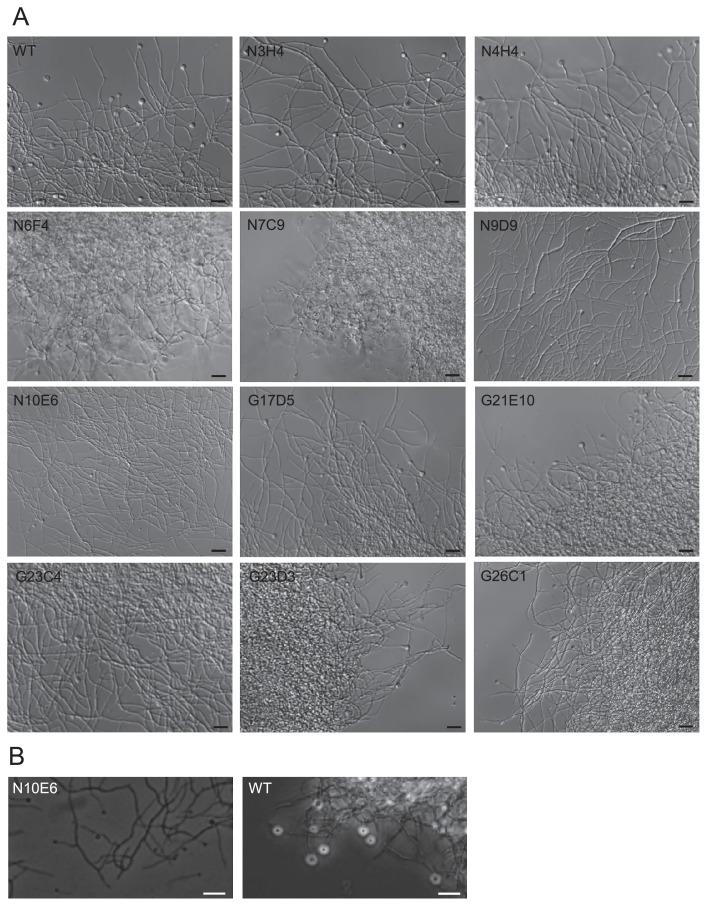
Microscopic images of hyphae and vesicles. (A) Differential interference contrast (DIC) and (B) phase-contrast images. Bars represent 10 μm.

The mutants N7C9, N10E6, G21E10, G23C4, and G23D3 produced significantly lower numbers of vesicles than the parental WT, while the mutants N4H4, G17D5, and G26C1 formed similar numbers of vesicles to the WT (Fig. 2). Vesicle size was found to differ with the mutant strains, and three classes were identified based on size. The mutants N6F4, N7C9, and N10E6 generated visibly smaller-sized vesicles, while the mutants N9D9, G17D5, G21E10, G23C4, G23D3, and G26C1 produced slightly smaller structures (Fig. 3A). In the mutants N3H4 and N4H4, vesicle size was similar to the parental WT (Fig. 3A). When observed under phase-contrast microscopy, vesicles of the mutant N10E6 had a phase dark appearance, in contrast to the phase bright appearance of mature vesicles found on the parental WT (Fig. 3B). Mutant strains with the aberrant vesicle phenotypes also often showed an abnormal hyphal morphology. The mutants N6F4, N7C9, G21E10, G23D3, and G26C1 had highly branched hyphae and formed condensed clumps, while the mutants N9D9 and N10E6 had less branched hyphae. The mutant N10E6 produced thinner hyphae than usual. When grown in liquid medium, the mutants N9D9 and N10E6 did not form hyphal clumps, which are characteristic for *Frankia* (data not shown).

### Expression of genes related to N_2_ fixation

**Fig. 4:**
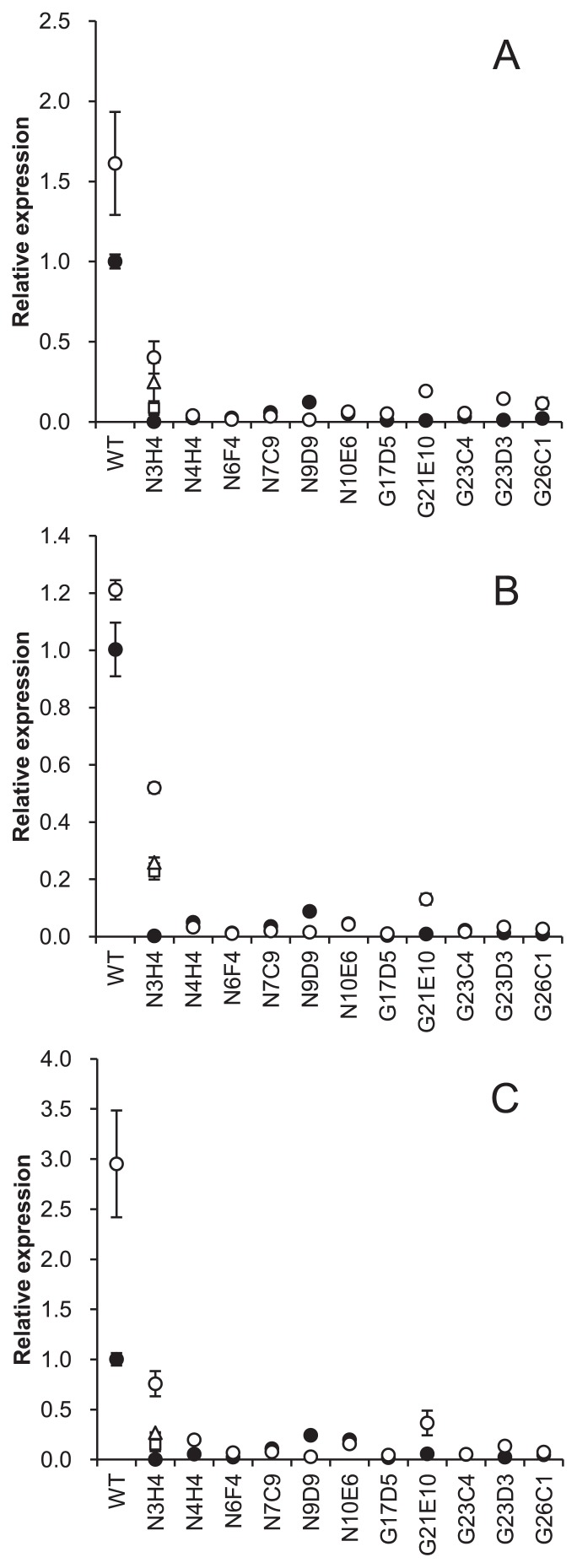
Expression of transcripts of *nifE* (A), *nifH* (B), and *nifV* (C). Relative levels to a value obtained from the wild-type (WT) sample are shown. Two to four RNA samples from different cultures were tested for each strain and results from the same RNA are indicated by the same symbol. The bar represents the standard deviation of 3 technical replicates.

The expression levels of *nifE* (biosynthetic scaffold for the FeMo co-factor) (10), *nifH* (dinitrogenase reductase), and *nifV* (homocitrate synthase) genes were estimated by detecting changes in mRNA levels using qRT-PCR. Under N- conditions, the mRNA levels of the *nifE*, *nifH*, and *nifV* genes were lower in all of the mutant strains than in the WT (Fig. 4). In particular, mutant strains N6F4, N7C9, G17D5, and G23C4 exhibited marked reductions in the mRNA levels of the three genes (less than 10% of the WT). Although variability among biological replicates was large, mutant strain N3H4 also exhibited reduced mRNA levels, whereas the levels of expression for these genes were higher than in the other mutant strains (Fig. 4).

### Genome analysis

The genomes of the six NTG-derived and two GR-derived mutants were resequenced, and found to contain 102 to 378 (NTG mutants) and 24 to 32 (GR mutants) mutations (Table 3). A large portion (≥90%) of the mapped reads for most of the mutations detected displayed a mutated genotype. Our only exception was strain N6F4, which had lower read coverage and we only detected 3 such reliable mutations. Notably, all eight strains carried bases at which all reads mapped on them displayed a mutant genotype (Table 3, 100%). This indicated that each of these strains consisted of clonal mutant cells. We checked for mutations in the known N_2_ fixation-related genes. Seven mutants carried a 9-bp deletion in the *hypF2* gene (Francci3_1072) encoding a hydrogenase maturation protein (Tables 3 and S2). In the WT and five mutant strains, we amplified the locus by PCR and confirmed the nucleotide sequence by the Sanger method (23). All of the strains carried a 9-bp deletion, but the position was different (5 bp downstream) from that detected by next-generation genome sequencing (Fig. S1). Strain N10E6 carried an amino acid change (Glu to Lys) in the putative squalene/phytoene dehydrogenase (Francci3_0821), which is assumed to be involved in the synthesis of hopanoid lipids, constituents of the vesicle envelope (5) (Tables 3 and S2). Strain N7C9 carried amino acid changes in Francci3_1942 (uptake hydrogenase large subunit, *hupL1*), Francci3_4487 (nitrogenase alpha subunit, *nifD*), and Francci3_4488 (nitrogenase reductase, *nifH*).

## Discussion

In multicellular bacteria such as *Frankia*, difficulties are associated with isolating loss-of-function mutants because a single colony formed from a mutagenized hyphae fragment typically contains mutant and WT cells and a recessive phenotype of the mutant cells is masked by a dominant WT phenotype. We previously developed the FF method to purify hyphae fragments consisting of clonal mutant cells and isolated a N_2_ fixation mutant using ethyl methanesulfonate (EMS) as a mutagen (13). In the present study, we tested two additional mutagens—NTG and GR—to isolate more N_2_ fixation mutants of *F. casuarinae* CcI3. We found that the type of mutagen resulted in different occurrence rates for mutants. When EMS was used, the occurrence rate was very low (0.04%, only one mutant/2,400 colonies). The phenotype of this mutant was not clear and ARA was not completely abolished (13). The use of NTG resulted in a 20-fold higher occurrence rate (0.8%, Table 1) than EMS and this was the most effective mutagen tested. Consistent with these results, NTG-mutagenized *Frankia* cells carried more mutations (100 to 400 per genome, Table 3) than EMS-mutagenized cells (9 to 20 per genome) (13). GR yielded slightly lower mutant-occurrence rates (0.6%, Table 1) than NTG. However, many of the NTG-derived strains had slow growth rates, and were growing very slowly, even under N+ conditions (data not shown). This growth deficiency may be attributed to the high numbers of accumulated mutations in NTG-mutagenized genomes (Table 3). A low mutation rate of EMS is advantageous for the identification of the mutation responsible for an aberrant phenotype; however, it is extremely labor-intensive to collect mutants with a clear phenotype. Therefore, GR appears be the best choice for the mutagenesis of *Frankia* cells. Of the 12 candidate mutant strains selected, 11 were genuine N_2_ fixation mutants (Fig. 1 and Table 2). This low false positive rate verifies the reliability of the FF method to enrich clonal mutant hyphae.

Due to the lack of a stable transformation system in *Frankia*, we were unable to identify the mutations (genes) responsible for aberrant phenotypes using a genetic complementation test. Therefore, we analyzed the genomes of the mutants and predicted the responsible mutations (Tables 3 and S2). Mutant strain N7C9 carried mutations in the *hupL1* (hydrogenase), *nifD*, and *nifH* genes (Table S2). The mutation in *hupL1* does not appear to be responsible for its complete loss of N_2_-fixing ability because mutants of hydrogenase in other diazotrophs retained strong N_2_-fixing abilities (11, 16). The mutant N7C9 carried one and two amino acid substitutions in NifD (Thr285Ile) and NifH (Ala61Val and Glu115Lys), respectively (Table S2). We mapped these amino acids in the crystal structure of the nitrogenase complex of *Azotobacter vinelandii* (24). His285 of *A. vinelandii* NifD (corresponding to Thr285 of WT *F. casuarinae*) and Glu116 of *A. vinelandii* NifH (Glu115 of WT *F. casuarinae*) were located on the outer surface of the nitrogenase complex and did not appear to make intimate contact with other amino acids (Fig. S2A). In contrast, Ala62 of *A. vinelandii* NifH (Ala61 of WT *F. casuarinae*) was located in close proximity to the NifD and NifK polypeptides, formed a hydrogen bond with Lys51 of NifD (Fig. S2B), and was involved in the interaction between NifDK and NifH. Collectively, these results indicate that the Ala61Val substitution in NifH causes the inability of the mutant N7C9 to fix N_2_.

Mutant strain N10E6 carried a mutation in a putative gene (Francci3_0821) encoding squalene/phytoene dehydrogenase (Table S2), which is involved in hopanoid lipid metabolism. The vesicles of mutant N10E6 were small and had a phase dark appearance (Fig. 3), indicating a decrease in the thickness of the vesicle envelope (22). These phenotypes suggest that the mutation in Francci3_0821 impaired the synthesis of hopanoid lipids, which are a constituent of the vesicle envelope, and then caused its aberrant vesicle differentiation, resulting in a N_2_-fixation defect under atmospheric oxygen levels. Since hopanoids also act to maintain membrane stability (25) and are contained in hyphae and vesicles (19), this mutation may cause the aberrant hyphal phenotypes observed in the strain.

The 9-bp deletion found in the *hypF2* gene (Francci3_1072) was not responsible for the inability to fix N_2_ because the parental WT strain carried it. Since the sequence is a part of tandem repeats (Fig. S1), it may have been deleted through homologous recombination. A deletion in such a repetitive sequence may not be detected correctly by next-generation sequencing. Genome analyses indicated that the phenotypes for most mutants were not caused by defects in any of the known N_2_ fixation-related genes (such as *nif*, *hup*, and *suf*) or the genes involved in hopanoid synthesis. Therefore, further research will identify novel genes involved in N_2_ fixation.

Although strains N3H4 and N6F4 shared most of the mutations (>100) in their genomes, the phenotype of the mutant N6F4 was more severe than that of the mutant N3H4. Since the two strains were isolated from the same mutagenized cell population (Table 1), it is highly possible that they originated from the same mutant cell. One explanation for this is that during the liquid culture prior to screening on solid media, they became separated and independently accumulated mutations, possibly by erroneous DNA replications.

Based on the lengths of intergenic regions (20), the *nifE*, *nifH*, and *nifV* genes belong to distinct operons in *F. casuarinae*. Nevertheless, transcripts of the three genes were always reduced in a similar manner in either of the mutants (Fig. 4). Mutant strains that showed a moderate (N3H4) or marked (including N6F4 and N7C9) reduction in *nifE* mRNA levels also showed a similar reduction in *nifH* and *nifV* mRNA levels. This result suggests that operons containing each gene are under the control of the same regulatory cascade. An unexpected result is that most (10 out of 11) of the mutants showed a significant reduction in *nif* transcripts (Fig. 4). Therefore, various mutations in genes other than canonical regulator proteins may affect transcriptional activation under N- conditions in *Frankia*.

Strains with aberrant vesicle phenotypes often showed abnormal hyphal branching patterns (Fig. 3A). This result suggests that genes regulating hyphal branching play a role in vesicle differentiation. Since vesicles often form at the tip of branched hyphae, this idea appears to be plausible.

Based on the levels of *nif* gene expression and vesicle formation, mutants were grouped into three classes. Class 1 mutants showed defects in vesicle formation and *nif* expression (N6F4, N7C9, N10E6, G21E10, G23C4, and G23D3). They appear to have a mutation in the genes responsible for vesicle differentiation and, as a consequence, the transcription of *nif* genes, which is regulated for exclusive expression in vesicle cells (17), did not occur. Alternatively, a master regulator gene(s) that simultaneously triggers vesicle differentiation and *nif* gene activation may be mutated. Class 2 mutants did not show any apparent defects in vesicle formation, but exhibited a severe reduction in *nif* gene expression (N4H4, N9D9, G17D5, and G26C1). In mutants of this class, it is highly possible that genes specifically required for the transcriptional activation of *nif* genes are mutated. The class 3 mutant did not show any apparent defects in vesicle formation or *nif* expression. Only strain N3H4 was categorized in this mutant class. Defects in several functions (*e.g.*, the import of metals (Mo and Fe), expression of *nif* genes other than *nifE*, *nifH*, and *nifV*, and permeability of oxygen through the vesicle envelope) may explain the phenotype; however, further research is needed in order to reach a more concrete conclusion.

## Supplementary Material



## Figures and Tables

**Table 1:** Summary of mutant screening.

Mutagen	Condition	Survival rate (%)	No. of colonies screened	No. of mutants^a^ (occurrence %)	Strains used in this study
NTG	1 mg mL^−1^, 4 min	—b	1248	5 (0.4)	
	1 mg mL^−1^, 7 min	—	846	7 (0.8)	
	1 mg mL^−1^, 10 min	11	480	9 (1.9)	N9D9, N10E6
	2 mg mL^−1^, 10 min	15	384	0 (0)	
	1 mg mL^−1^, 20 min	0.8	576	9 (1.6)	N3H4, N4H4, N6F4, N7C9
	Total		3534	30 (0.8)	
GR	772 Gy	39	2168	6 (0.3)	G1G7, G17D5, G21E10
	1158 Gy	5	1080	13 (1.2)	G23C4, G23D3, G26C1
	Total		3248	19 (0.6)	

a Mutants that showed a clear phenotype in the second screening.

b Not determined.

**Table 2:** ARA of mutants.

Strain	Days after transfer to N- conditions				

	5	6	7	8	9
WT	15±6.5	54±27	12±4.3	16±10	17±8.9
N3H4	ND^a^	ND	ND	ND	ND
N4H4	ND	ND	ND	ND	ND
N6F4	ND	ND	ND	ND	ND
N7C9	ND	ND	ND	ND	ND
N9D9	ND	ND	ND	ND	ND
N10E6	ND	ND	ND	ND	ND
G1G7	46±13	11±11	ND	ND	ND
G17D5	—b	ND	ND	ND	ND
G21E10	—	ND	ND	ND	ND
G23C4	—	ND	ND	ND	ND
G23D3	—	ND	ND	ND	ND
G26C1	—	ND	ND	ND	ND

ARA is represented as nmol ethylene h^−1^ mg protein^−1^. Values are means with standard errors calculated from 3 to 4 replicates.

a Not detected.

b Not determined.

**Table 3:** Summary of the genome analysis.

Strain	Average depth^a^	No. of mutations			

		Total	≥90%^b^	100%^c^	N_2_ fixation^d^
N3H4	1500	117	104	4	1
N4H4	1463	102	90	4	1
N6F4	342	116	3	2	1
N7C9	1477	223	220	6	5
N9D9	1736	273	248	9	1
N10E6	1792	378	372	10	2
G21E10	157	24	22	21	0
G23C4	123	32	32	23	1

a Average number of reads mapped on mutated bases.

b 90% or more of mapped reads displayed a mutant genotype.

c 100% of mapped reads displayed a mutant genotype. Maximum depths at the mutations were 1693 (N3H4), 1491 (N4H4), 353 (N6F4), 1502 (N7C9), 1731 (N9D9), 2064 (N10E6), 192 (G21E10), and 186 (G23C4).

d Mutations found in N_2_ fixation-related genes. Only those with changed amino acid sequences are included.
