# Supplementary material for: Nitrogen Fixation Mutants of the Actinobacterium Frankia Casuarinae CcI3
Source: Microbes Environ. 2017 Nov 18;32(4):344–51. doi: 10.1264/jsme2.ME17099 (PMC5745019; doi:10.1264/jsme2.ME17099)
Supplement: Supplementary file 1 [file 32_344_s1.pdf]

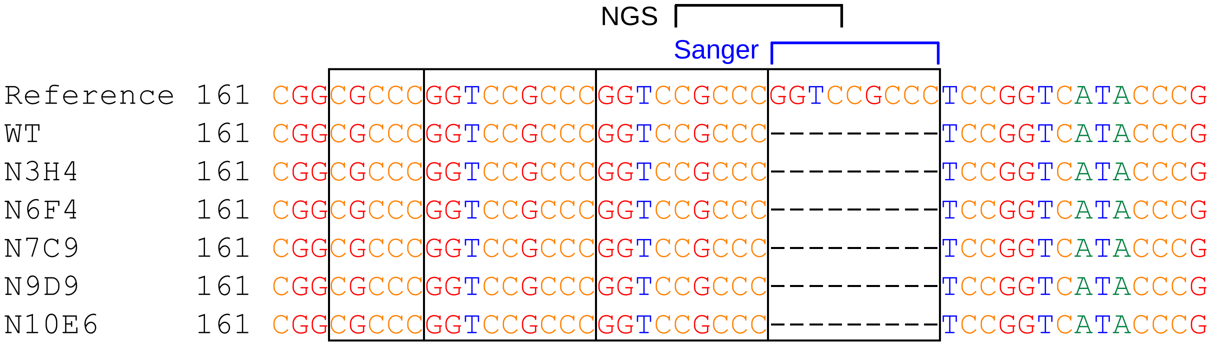

Fig. S1. Nucleotide sequence of the *hypF2* gene (Francci3_1072). Repetitive sequences are boxed. Deletions detected by the next-generation sequencing (black bracket) and the Sanger sequence (blue bracket) are shown.

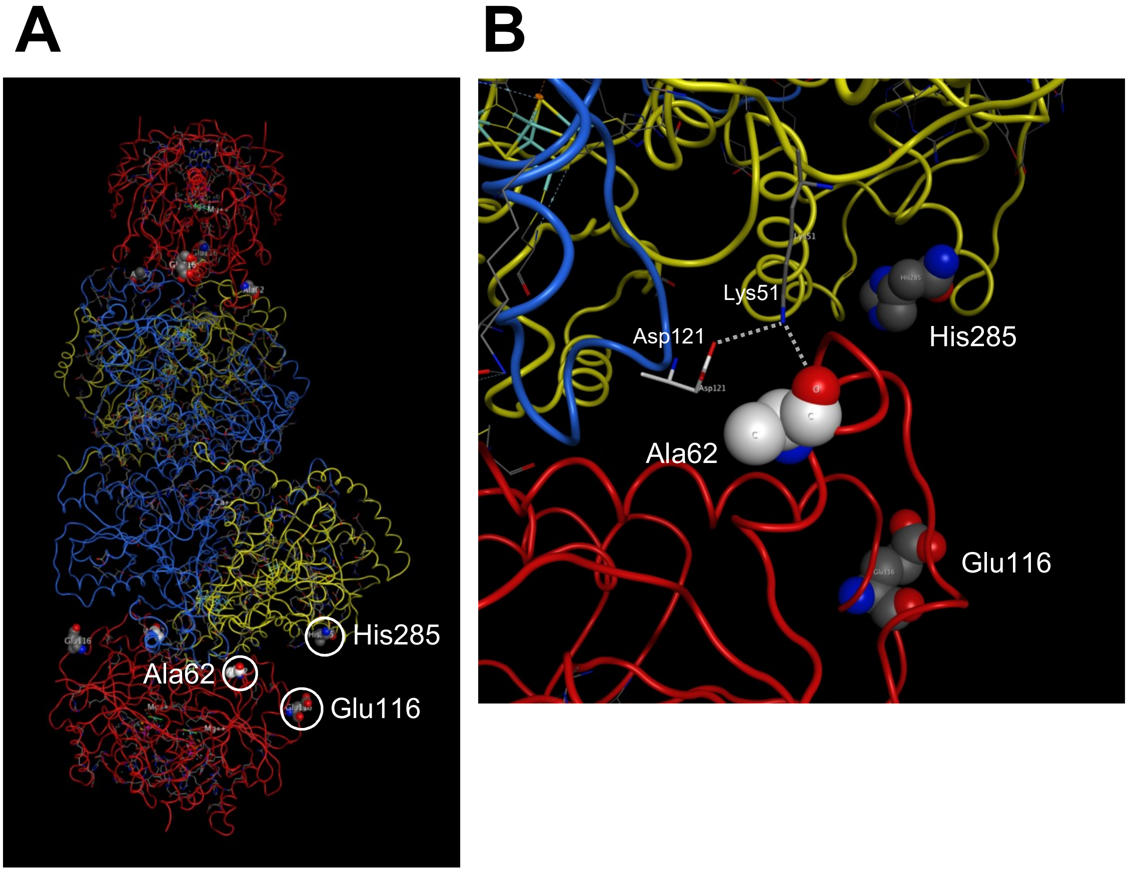

Fig. S2. Crystal structure of the nitrogenase complex of *Azotobacter vinelandii* (PDB ID: 1N2C). Yellow, blue and red polypeptides represent NifD, NifK and NifH, respectively. Overall structure (A) and a magnified view (B) are shown. Hydrogen bonds are represented as dashed gray line.

Table S1. Primers and TaqMan probes used in quantitative reverse-transcription PCR

Gene (ID)	Usage[a]	Sequence (5' to 3')
16S rRNA (Francci3_R0040)	F	cggtgaaatgcgcagatatcag
	R	gtggactaccagggtatctaatcct
	T	aaagcgtggggagcga
nifE (Francci3_4485)	F	cgacgcaaaaatgatcaaag
	R	gtaagggatatgccgctcct
	T	tgccgttcctcgatatcaac
nifH (Francci3_4488)	F	agatgggcaagaaggtcatgatc
	R	aggtctgggccttggaatg
	T	actcgacccggctcatcc
nifV (Francci3_4489)	F	ttcaccgcagccgagaa
	R	aggtattccggcctcgatct
	T	ctgctctggacgccatcg

[a]F, forward primer; R, reverse primer; T, TaqMan probe

Table S2. Mutations found in mutant genomes

Position (nt)	Mutation type*	WT allele	Mutant allele	Gene ID	Annotation	N fixation related	Amino acid change	Reads that called a mutation/all reads (%)							
								N3H4	N4H4	N6F4	N7C9	N9D9	N10E6	G21E10	G23C4
635	SNV	G	A	Francci3_0001	chromosomal replication initiator protein DnaA		Val201Ile				99.7				
3,774	SNV	C	T	Francci3_0004	recombination protein F		Ala26Val	100		80.4					
18,176	SNV	C	T										99.8		
28,054	SNV	C	T					99.8		76.5					
29,793	SNV	G	A	Francci3_0021	CRISPR-associated Cas5e family		Ala196Thr						99.3		
29,864	SNV	G	A	Francci3_0021	CRISPR-associated Cas5e family								99.7		
29,901	SNV	C	T	Francci3_0021	CRISPR-associated Cas5e family		Pro232Ser	99.7		83.9					
30,042	SNV	G	A										99.9		
30,413	SNV	C	T	Francci3_0022	hypothetical protein		Thr123Ile	99.1		80.9					
33,165	SNV	G	C	Francci3_0027	hypothetical protein		Arg76Pro								100
33,166	SNV	G	T	Francci3_0027	hypothetical protein										98.8
33,167	SNV	G	C	Francci3_0027	hypothetical protein		Gly77Arg								100
33,168	SNV	G	A	Francci3_0027	hypothetical protein		Gly77Glu								100
33,177	SNV	C	G	Francci3_0027	hypothetical protein		Ser80Trp								100
33,180	SNV	C	A	Francci3_0027	hypothetical protein		Ala81Glu								100
33,181	SNV	G	A	Francci3_0027	hypothetical protein										100
33,182	SNV	G	A	Francci3_0027	hypothetical protein		Gly82Ser								100
33,187	SNV	G	T	Francci3_0027	hypothetical protein										98.9
33,192	SNV	A	C	Francci3_0027	hypothetical protein		Gln85Pro								100
33,194	SNV	T	G	Francci3_0027	hypothetical protein		Trp86Gly								98.8
39,697	SNV	G	A	Francci3_0033	PBP family phospholipid-binding		Ser101Asn					99.8			
54,017	SNV	C	T	Francci3_0044	serine/threonine protein kinase		Ser668Asn					99.8			
59,143	SNV	G	A	Francci3_0047	hypothetical protein		Trp95*				99.7				
70,043	SNV	G	A	Francci3_0054	hypothetical protein		Gly26Glu		99.6						
81,769	SNV	G	A								99.9				
90,047	SNV	C	T	Francci3_0071	hypothetical protein		Pro223Ser	99.7		79.9					
92,554	SNV	G	A	Francci3_0073	hypothetical protein		Ala56Val				99.8				
101,161	SNV	C	T	Francci3_0082	phosphoserine aminotransferase		Thr365Met						99.8		
108,757	SNV	T	C						99.8						
114,902	SNV	C	T	Francci3_0093	hypothetical protein		Pro197Ser						99.7		
118,225	SNV	C	T						99.4						
120,352	SNV	G	A	Francci3_0098	hypothetical protein							99.7			
133,561	SNV	G	A	Francci3_0110	putative DNA helicase		Ala394Thr		99.8						
136,719	SNV	C	T	Francci3_0112	B12-dependent methionine synthase				99.8						
140,582	SNV	C	T	Francci3_0114	phage integrase		Gly340Asp					99.8			
145,620	SNV	C	T	Francci3_0119	signal transduction protein				99.8						
145,716	SNV	C	T	Francci3_0119	signal transduction protein				99.9						
152,467	SNV	G	A	Francci3_0122	transposase, IS4						99.3				

Table S2

Position (nt)	Mutation type*	WT allele	Mutant allele	Gene ID	Annotation	N fixation related	Amino acid change	Reads that called a mutation/all reads (%)							
								N3H4	N4H4	N6F4	N7C9	N9D9	N10E6	G21E10	G23C4
152,742	SNV	C	T						98.6						
156,411	SNV	G	A	Francci3_0126	hypothetical protein				99.7						
161,682	SNV	C	T									93.8			
163,851	SNV	C	T									93.6			
164,759	SNV	C	T									94.9			
164,897	SNV	C	T									94.6			
165,376	SNV	C	T									93.6			
166,179	SNV	C	T	Francci3_0138	cell divisionFtsK/SpoIIIE		His54Tyr					94.9			
167,381	SNV	C	T	Francci3_0138	cell divisionFtsK/SpoIIIE							94.6			
168,146	SNV	C	T	Francci3_0139	hypothetical protein							97.7			
172,683	SNV	G	A	Francci3_0144	transposase IS66		Ala264Thr					99.6			
183,527	SNV	G	A	Francci3_0156	hypothetical protein		Ala275Val					94.2			
215,363	SNV	C	T	Francci3_0183	L-threonine aldolase			99.8		80.3					
229,703	SNV	C	T	Francci3_0194	hypothetical protein		Ala383Val		99.8						
242,254	SNV	C	T	Francci3_0204	putative O-methyltransferase		Gly62Ser		99.8						
244,477	SNV	C	T								99.5				
246,858	SNV	G	A	Francci3_0208	XRE family transcriptional regulator				99.9						
248,324	SNV	G	A	Francci3_0210	MarR family transcriptional regulator		Arg203Trp		99.6						
270,916	SNV	C	T								99.6				
277,100	SNV	C	T	Francci3_0236	HemK family modification methylase		Ala314Val					99.7			
280,486	SNV	C	T	Francci3_0241	ABC transporter related		Arg131Cys						99.9		
287,597	SNV	C	T	Francci3_0249	ABC transporter related						99.9				
288,877	SNV	G	A	Francci3_0250	binding-protein-dependent transport systems inner membrane component								100		
289,805	SNV	G	A	Francci3_0251	putative sulfonate binding protein		Ala150Val						99.7		
305,465	SNV	C	T					99.8		81.2					
333,671	SNV	G	A	Francci3_0287	magnesium and cobalt transport protein CorA		Ser84Phe					99.6			
334,014	SNV	C	T					99.9		78.8					
337,585	SNV	G	A	Francci3_0291	multi-sensor signal transduction histidine kinase		Glu135Lys					99.9			
337,733	SNV	G	A	Francci3_0291	multi-sensor signal transduction histidine kinase		Arg184His					99.5			
339,372	SNV	G	A	Francci3_0292	hypothetical protein							100			
344,203	SNV	G	A	Francci3_0296	insertion element hypothetical protein		Pro198Leu	99.6		72.0					
356,941	SNV	G	A	Francci3_0309	periplasmic sensor signal transduction histidine kinase		Ala444Thr				99.5				
362,442	SNV	G	A	Francci3_0314	amidohydrolase 2		Ala119Val					99.7			
365,607	SNV	C	T	Francci3_0316	saccharopine dehydrogenase		Gly115Ser						99.9		
366,277	SNV	G	A	Francci3_0317	pyruvate carboxylase		Gly81Ser		99.6				99.6	99.4	100
369,854	SNV	C	T	Francci3_0318	glycosyl transferase family protein				76.8						

Table S2

Position (nt)	Mutation type*	WT allele	Mutant allele	Gene ID	Annotation	N fixation related	Amino acid change	Reads that called a mutation/all reads (%)							
								N3H4	N4H4	N6F4	N7C9	N9D9	N10E6	G21E10	G23C4
374,084	SNV	C	T	Francci3_0320	periplasmic sensor signal transduction histidine kinase		Glu407Lys	99.5		78.9					
374,268	SNV	G	A	Francci3_0320	periplasmic sensor signal transduction histidine kinase			99.7		77.6					
386,869	SNV	G	A	Francci3_0330	MarR family transcriptional regulator		Arg61Trp		99.9						
388,918	SNV	G	A	Francci3_0332	metallophosphoesterase			100		76.7					
392,523	SNV	G	A	Francci3_0338	L-asparaginase II		Gly205Glu				99.2				
455,633	SNV	C	T	Francci3_0382	amino acid/amide ABC transporter ATP-binding protein										99.3
461,677	SNV	G	A										99.8		
465,228	SNV	C	T	Francci3_0393	recombinase				99.7						
485,601	SNV	C	T	Francci3_0409	peptidoglycan-binding LysM		Glu69Lys		99.8						
486,381	SNV	C	T						99.7						
488,602	SNV	C	T	Francci3_0412	cell divisionFtsK/SpoIIIE		Asp1093Asn					99.4			
490,557	SNV	G	A	Francci3_0412	cell divisionFtsK/SpoIIIE		Ser441Phe					99.9			
491,451	SNV	C	T	Francci3_0412	cell divisionFtsK/SpoIIIE		Arg143Gln	99.9		78.7					
491,695	SNV	G	A	Francci3_0412	cell divisionFtsK/SpoIIIE				99.8						
494,891	SNV	C	T								99.8				
548,093	SNV	G	A						99.9						
551,806	SNV	C	T										99.8		
565,885	SNV	G	A	Francci3_0484	redox-sensing transcriptional repressor Rex		Asp56Asn				99.9				
568,049	SNV	G	A	Francci3_0485	glutamyl-tRNA reductase							99.8			
572,439	SNV	G	A								99.4				
572,837	SNV	G	A									99.4			
573,496	SNV	G	A	Francci3_0490	heavy metal translocating P-type		Ala74Thr					99.9			
576,885	SNV	G	A	Francci3_0491	methionyl-tRNA synthetase		Arg192Cys						99.9		
579,072	SNV	G	A								99.7				
579,243	SNV	G	A								99.6				
579,375	SNV	C	T	Francci3_0494	hypothetical protein							99.5			
579,956	SNV	G	A	Francci3_0494	hypothetical protein		Gly201Asp				99.2				
581,183	SNV	G	A	Francci3_0496	integrase		Gly217Arg					99.5			
590,244	SNV	G	A									99.8			
591,926	SNV	G	A	Francci3_0506	hypothetical protein							99.6			
606,917	SNV	C	T	Francci3_0522	hypothetical protein		Thr3Ile					99.8			
613,269	SNV	C	T	Francci3_0528	uroporphyrinogen-III C-						99.5				
618,959	SNV	G	C						99.5		99.6	99.6	99.6		
626,021	SNV	G	A	Francci3_0539	NADH dehydrogenase subunit B		Ala226Thr						99.5		
641,339	SNV	C	T	Francci3_0551	NADH dehydrogenase subunit N		Ala429Val				99.9				
649,890	SNV	G	A										99.8		

Table S2

Position (nt)	Mutation type*	WT allele	Mutant allele	Gene ID	Annotation	N fixation related	Amino acid change	Reads that called a mutation/all reads (%)							
								N3H4	N4H4	N6F4	N7C9	N9D9	N10E6	G21E10	G23C4
649,919	SNV	G	A										99.7		
651,803	SNV	G	A	Francci3_0560	AIG2-like		Gly89Ser						99.9		
664,551	SNV	G	A	Francci3_0573	DNA-directed RNA polymerase subunit beta		Gly518Asp					99.7			
664,598	SNV	G	A	Francci3_0573	DNA-directed RNA polymerase subunit beta		Val534Ile					99.6			
683,805	SNV	C	T									99.9			
691,339	SNV	G	A	Francci3_0606	50S ribosomal protein L36		Gly21Glu		99.7						
694,215	SNV	G	A	Francci3_0610	DNA-directed RNA polymerase subunit alpha		Arg287His				99.9				
700,697	SNV	G	A	Francci3_0617	glucosamine--fructose-6-phosphate aminotransferase		Gly49Asp					99.6			
707,850	SNV	C	T	Francci3_0623	hypothetical protein		Ala66Thr						99.5		
709,091	SNV	G	A	Francci3_0624	serine hydroxymethyltransferase							99.6			
709,707	SNV	G	A										99.6		
710,306	SNV	C	T	Francci3_0625	alpha/beta hydrolase fold		Pro149Leu				99.4				
710,527	SNV	C	T	Francci3_0625	alpha/beta hydrolase fold		Pro223Ser				99.6				
711,347	SNV	C	T	Francci3_0626	hypothetical protein						99.4				
712,128	SNV	C	T	Francci3_0627	peptidase M22, glycoprotease						99.7				
716,001	SNV	G	A	Francci3_0633	chaperonin GroEL		Val106Met						100		
724,312	SNV	C	T	Francci3_0639	hypothetical protein		Ala71Val					99.9			
733,743	SNV	G	A								99.7				
740,991	SNV	G	A								99.5				
759,686	SNV	C	T	Francci3_0663	ribonuclease BN		Leu271Phe						100		
763,614	SNV	C	T	Francci3_0666	GCN5-related N-acetyltransferase		Trp50*						99.6		
773,372	SNV	C	T	Francci3_0677	ATP-dependent DNA helicase RecQ		Ala321Val				99.7				
774,436	SNV	G	A	Francci3_0677	ATP-dependent DNA helicase RecQ		Val676Ile					100			
775,543	SNV	C	T	Francci3_0679	succinate dehydrogenase flavoprotein subunit		Asp535Asn				99.6				
777,966	SNV	C	T	Francci3_0681	succinate dehydrogenase, cytochrome b subunit		Val29Ile				99.9				
780,757	SNV	C	T	Francci3_0683	serine/threonine protein kinase		Asp584Asn				99.8				
786,665	SNV	C	T	Francci3_0688	deoxyribose-phosphate aldolase		Gly188Asp				99.4				
827,044	SNV	G	A								99.6				
845,101	SNV	G	A	Francci3_0730	glycosyl transferase family protein		Gly29Glu						99.9		
851,890	SNV	C	T	Francci3_0736	glycosyl transferase family protein							100			
859,601	SNV	C	T	Francci3_0742	LPPG:FO 2-phospho-L-lactate		Gly17Ser							100	99.3
860,482	SNV	C	T	Francci3_0743	TetR family transcriptional regulator		Arg2His		99.6						
864,442	SNV	C	T	Francci3_0746	transcription factor WhiB		Thr61Ile		99.7						
867,278	SNV	C	T	Francci3_0747	glycosyltransferases-like						99.8				

Table S2

Position (nt)	Mutation type*	WT allele	Mutant allele	Gene ID	Annotation	N fixation related	Amino acid change	Reads that called a mutation/all reads (%)							
								N3H4	N4H4	N6F4	N7C9	N9D9	N10E6	G21E10	G23C4
870,968	SNV	G	A	Francci3_0748	mannose-6-phosphate isomerase		Ala179Val					99.7			
875,338	SNV	G	A	Francci3_0753	hypothetical protein		Asp268Asn						99.8		
885,477	SNV	G	A	Francci3_0760	hypothetical protein		Pro163Leu						99.6		
888,996	SNV	G	A										99.8		
892,625	SNV	G	A	Francci3_0767	preprotein translocase subunit SecA								99.9		
896,288	SNV	C	T	Francci3_0769	hypothetical protein		Gly218Asp						99.3		
898,595	SNV	G	A	Francci3_0772	hypothetical protein		Gly121Glu						99.9		
899,224	SNV	G	A	Francci3_0773	chromosome partitioning ATPase protein-like		Gly74Asp					99.7			
899,328	SNV	G	A	Francci3_0773	chromosome partitioning ATPase protein-like		Val109Ile						99.8		
904,727	SNV	G	A	Francci3_0777	hypothetical protein								99.8		
906,938	SNV	C	T										99.8		
907,746	SNV	G	A	Francci3_0779	type II secretion system protein E		Val120Ile						99.6		
908,223	SNV	G	A	Francci3_0779	type II secretion system protein E		Asp279Asn						99.7		
916,069	SNV	C	T	Francci3_0786	peptidase S15		Gly514Asp					100			
917,696	SNV	G	A	Francci3_0787	hypothetical protein								99.9		
926,855	SNV	G	A	Francci3_0794	assimilatory nitrite reductase (NAD(P)H) large subunit precursor		Ala618Thr						99.8		
933,268	SNV	C	T	Francci3_0801	SsrA-binding protein		Thr99Ile							100	
936,706	SNV	C	T	Francci3_0804	hypothetical protein		Arg2Gln	99.8		84.1					
939,241	SNV	C	T					99.5		76.4					
942,102	SNV	C	T	Francci3_0810	resolvase helix-turn-helix region		Gly31Asp						99.6		
944,727	SNV	C	T	Francci3_0813	hypothetical protein		Pro278Ser						99.8		
947,895	SNV	C	T										99.8		
948,208	SNV	C	T										99.9		
948,444	SNV	C	T	Francci3_0815	NUDIX hydrolase		Pro34Ser						99.9		
958,219	SNV	G	A	Francci3_0821	putative squalene/phytoene dehydrogenase (spd), FRAAL1430	yes	Glu425Lys						99.9		
972,847	SNV	G	A	Francci3_0832	urease subunit alpha							99.9			
989,301	Deletion	CCCGGGGTG	-	Francci3_0847	hypothetical protein		Pro27_Thr29 del		68.6		67.8	67.1	67.9		
998,973	Deletion	CGGCTGG	-					85.6		69.6					
1,021,021	SNV	C	T	Francci3_0876	putative ATP/GTP binding protein		Arg203His					99.7			
1,022,313	SNV	G	A	Francci3_0877	DNA integration/recombination/invertion		Ala115Thr	99.9		80.0					
1,025,191	SNV	C	T	Francci3_0880	antirestriction protein			99.6		84.3					
1,033,703	SNV	C	T										99.8		

Table S2

Position (nt)	Mutation type*	WT allele	Mutant allele	Gene ID	Annotation	N fixation related	Amino acid change	Reads that called a mutation/all reads (%)							
								N3H4	N4H4	N6F4	N7C9	N9D9	N10E6	G21E10	G23C4
1,033,729	SNV	C	T										99.9		
1,047,325	SNV	G	A										99.9		
1,050,093	SNV	G	A	Francci3_0906	Type IV secretory pathway VirD4 components-like								99.6		
1,051,590	SNV	C	T	Francci3_0907	hypothetical protein		Ala98Val				99.7				
1,053,590	SNV	G	A	Francci3_0910	recombinase				99.9						
1,066,859	SNV	C	T	Francci3_0923	ATP-dependent transcription regulator LuxR		Pro297Ser	56.3							
1,070,540	SNV	G	A									99.7			
1,101,724	SNV	G	A	Francci3_0937	pyridine nucleotide-disulphide oxidoreductase dimerisation region		Arg245Gln	51.6							
1,103,975	SNV	C	T	Francci3_0938	FAD dependent oxidoreductase		Gly122Asp					99.7			
1,115,956	SNV	G	A	Francci3_0947	FHA domain-containing protein					79.9					
1,121,055	SNV	C	T	Francci3_0949	hypothetical protein		Gly201Glu						99.9		
1,130,852	SNV	G	A	Francci3_0956	hypothetical protein		Pro5Ser						99.9		
1,135,739	SNV	G	A	Francci3_0962	D-lactate dehydrogenase								99.4		
1,136,325	SNV	C	T	Francci3_0962	D-lactate dehydrogenase							99.9			
1,136,619	SNV	G	A	Francci3_0962	D-lactate dehydrogenase		Ala555Thr						99.0		
1,140,250	SNV	G	A	Francci3_0964	glycosyl transferase family protein		Val340Ile						99.3		
1,144,065	SNV	C	T	Francci3_0966	hypothetical protein								99.6		
1,150,728	SNV	G	A	Francci3_0973	methyltransferase type 11			99.8		82.7					
1,152,090	SNV	G	A	Francci3_0974	hypothetical protein			99.4		79.5					
1,153,235	SNV	G	A	Francci3_0975	NADH dehydrogenase		Arg237Trp						99.6		
1,155,430	SNV	C	T	Francci3_0976	NAD-dependent		Val70Ile	99.3		81.9					
1,165,765	SNV	G	A						99.7						
1,167,194	SNV	G	A						99.5						
1,168,624	SNV	C	T	Francci3_0988	carboxyl transferase		Glu596Lys	99.8		83.5					
1,168,755	SNV	C	T	Francci3_0988	carboxyl transferase		Gly552Asp					74.2			
1,187,021	SNV	G	A	Francci3_0997	alpha/beta hydrolase fold								99.6		
1,188,050	SNV	A	G	Francci3_0998	hypothetical protein		Ser116Pro						99.5		
1,188,579	SNV	C	T						99.6		99.3	99.5	99.7	100	100
1,194,828	SNV	C	T								99.7				
1,208,808	SNV	G	A	Francci3_1014	hypothetical protein						99.3				
1,209,586	SNV	G	A	Francci3_1015	putative DNA-binding protein		Ala84Thr				99.9				
1,217,386	SNV	C	T	Francci3_1022	XRE family transcriptional regulator								99.7		
1,221,346	SNV	C	T	Francci3_1028	cell divisionFtsK/SpoIIIE		Pro451Ser						99.7		
1,223,525	SNV	C	T	Francci3_1031	excisionase/Xis, DNA-binding		Thr2Ile						99.8		
1,225,243	SNV	G	A						99.8						
1,230,948	SNV	C	T	Francci3_1035	hypothetical protein		Pro74Leu						99.6		
1,232,854	SNV	C	T	Francci3_1037	polysaccharide deacetylase		Pro218Leu		99.8						

Table S2

Position (nt)	Mutation type*	WT allele	Mutant allele	Gene ID	Annotation	N fixation related	Amino acid change	Reads that called a mutation/all reads (%)							
								N3H4	N4H4	N6F4	N7C9	N9D9	N10E6	G21E10	G23C4
1,233,877	SNV	C	T	Francci3_1038	aldehyde dehydrogenase		Ala238Thr						99.7		
1,238,544	SNV	C	T	Francci3_1042	endopeptidase La		Gly128Asp					99.7			
1,254,681	SNV	G	A	Francci3_1054	acyltransferase 3						99.8				
1,278,183	Deletion	CGCC CGGT	-	Francci3_1072	hydrogenase maturation protein hypF2, FRAAL1825	yes	Thr12_Arg14 del	80.5	81.6	83.4	82.8	80.7	82.6		NA†
1,294,465	SNV	C	T	Francci3_1084	hypothetical protein		Gly321Asp				99.3				
1,309,941	SNV	G	A	Francci3_1097	hypothetical protein		Pro7Ser						99.7		
1,337,245	SNV	G	A									99.7			
1,341,384	SNV	G	A	Francci3_1127	lantibiotic dehydratase-like		Pro803Leu				99.8				
1,355,246	SNV	C	T	Francci3_1140	hypothetical protein		Arg43Cys					99.7			
1,367,403	SNV	C	T	Francci3_1150	alpha amylase, catalytic region		Ala385Thr				99.8				
1,368,037	SNV	C	T	Francci3_1150	alpha amylase, catalytic region						99.7				
1,369,297	SNV	C	T	Francci3_1151	serine phosphatase						99.7				
1,369,387	SNV	G	A	Francci3_1151	serine phosphatase								99.8		
1,383,035	SNV	C	T	Francci3_1160	Membrane alanyl aminopeptidase. Metallo peptidase. MEROPS family		Trp169*							100	98.7
1,411,329	SNV	C	T	Francci3_1180	exodeoxyribonuclease III		Ala286Thr	99.8		85.5					
1,417,995	SNV	G	A	Francci3_1185	dihydroorotate oxidase B, catalytic		Gly82Asp						99.8		
1,432,635	SNV	C	T	Francci3_1199	putative ATP-binding protein							99.8			
1,438,453	SNV	C	T								99.3				
1,456,880	SNV	G	A	Francci3_1218	ribonuclease		Glu540Lys						99.8		
1,470,337	SNV	C	T	Francci3_1227	gamma-glutamyl phosphate reductase		Pro402Ser				99.5				
1,471,752	SNV	C	T	Francci3_1228	malate synthase							99.9			
1,485,421	SNV	C	T	Francci3_1240	Poly(3-hydroxybutyrate)			99.4		78.6					
1,486,124	SNV	G	A	Francci3_1241	beta-lactamase-like		Ser149Asn						99.8		
1,496,332	SNV	G	A	Francci3_1251	alkane 1-monooxygenase							99.7			
1,501,569	SNV	G	A	Francci3_1257	helix-hairpin-helix DNA-binding, class		Ala336Thr					71.8			
1,556,650	SNV	C	T	Francci3_1301	glycosyl transferase family protein								99.9		
1,560,110	SNV	G	A	Francci3_1303	methyltransferase FkbM						99.9				
1,574,610	SNV	C	T	Francci3_1312	aconitase		Ala809Val	99.4		84.2					
1,575,495	SNV	C	T	Francci3_1313	inositol-1(or 4)-monophosphatase		Ala198Thr						99.8		
1,579,460	SNV	C	T	Francci3_1315	major facilitator transporter							99.6			
1,582,403	SNV	C	T	Francci3_1318	deoxyuridine 5'-triphosphate nucleotidohydrolase							99.6			
1,585,174	SNV	G	A	Francci3_1322	TrkA-like			99.7		81.2					
1,585,376	SNV	G	A					99.5		85.0					
1,585,756	SNV	G	A	Francci3_1323	TrkA-like			99.8		85.8					
1,585,964	SNV	G	A	Francci3_1323	TrkA-like		Thr85Ile	99.8		79.1					
1,609,178	SNV	C	T	Francci3_1340	inositol monophosphatase		Thr11Ile					99.8			
1,611,095	SNV	C	T	Francci3_1342	sulfate adenylyltransferase subunit 1		Ser12Phe					99.8			

Table S2

Position (nt)	Mutation type*	WT allele	Mutant allele	Gene ID	Annotation	N fixation related	Amino acid change	Reads that called a mutation/all reads (%)							
								N3H4	N4H4	N6F4	N7C9	N9D9	N10E6	G21E10	G23C4
1,620,466	SNV	C	T	Francci3_1349	malto-oligosyltrehalose synthase			99.4		77.8					
1,645,607	SNV	C	T	Francci3_1371	Holliday junction DNA helicase RuvB		Ala93Val				99.6				
1,649,202	SNV	C	T	Francci3_1374	preprotein translocase subunit SecF				99.8		99.8	99.9	99.8	100	100
1,651,478	SNV	C	T	Francci3_1376	(p)ppGpp synthetase I		His164Tyr				100				
1,679,432	SNV	G	A	Francci3_1396	FAD dependent oxidoreductase						99.7				
1,685,555	SNV	G	A	Francci3_1399	hypothetical protein		Gly154Glu						99.7		
1,707,138	SNV	C	T					99.7		84.1					
1,742,951	SNV	G	A	Francci3_1451	hypothetical protein		Ala104Thr						98.7		
1,760,667	SNV	G	A	Francci3_1468	FHA domain-containing protein							99.5			
1,760,855	SNV	G	A	Francci3_1468	FHA domain-containing protein		Arg146His					99.6			
1,767,739	SNV	C	T	Francci3_1475	cell divisionFtsK/SpoIIIE							99.6			
1,769,207	SNV	C	T	Francci3_1475	cell divisionFtsK/SpoIIIE		Arg982Trp	99.9		79.8					
1,801,481	SNV	G	A	Francci3_1510	hypothetical protein				99.2						
1,838,181	SNV	C	T	Francci3_1532	periplasmic binding protein/LacI transcriptional regulator		Trp38*						94.9		
1,841,263	SNV	G	A	Francci3_1535	ATPase		Glu373Lys						99.7		
1,841,850	Deletion	CGCT GCCG CCGT	-	Francci3_1535	ATPase		Ala569fs	50.7							
1,846,150	SNV	G	A	Francci3_1537	MMPL							99.9			
1,855,382	SNV	G	A	Francci3_1546	GCN5-related N-acetyltransferase							99.9			
1,861,567	SNV	C	T	Francci3_1552	helicase RecD/TraA		Ser354Phe					99.8			
1,870,125	Deletion	G	-						82.8				80.7		
1,871,157	SNV	C	T	Francci3_1560	putative signal transduction histidine kinase		Ala365Thr				99.6				
1,873,160	SNV	C	T	Francci3_1561	4-hydroxybutyrate coenzyme A transferase		Ala270Val				99.7				
1,887,122	SNV	G	A	Francci3_1572	parallel beta-helix repeat-containing		Glu240Lys				99.6				
1,888,731	SNV	G	A	Francci3_1574	glycosyl transferase, group 1		Asp56Asn					99.8			
1,889,842	SNV	C	T	Francci3_1575	hypothetical protein						99.6				
1,895,643	SNV	C	T	Francci3_1580	glycosyl transferase family protein							100			
1,902,557	SNV	G	A	Francci3_1586	glycosyl transferase family protein		Pro284Ser					100			
1,908,488	SNV	A	G	Francci3_1591	binding-protein-dependent transport systems inner membrane component		Val145Ala						99.7		
1,916,673	SNV	G	A	Francci3_1596	hypothetical protein						99.9				
1,927,074	SNV	G	A	Francci3_1605	cyclopropane-fatty-acyl-phospholipid synthase						99.8				
1,948,128	SNV	C	T								99.7				
1,957,903	SNV	C	T	Francci3_1628	integral membrane protein TerC		Pro322Ser				99.2				
1,958,226	SNV	C	T								99.3				

Table S2

Position (nt)	Mutation type*	WT allele	Mutant allele	Gene ID	Annotation	N fixation related	Amino acid change	Reads that called a mutation/all reads (%)							
								N3H4	N4H4	N6F4	N7C9	N9D9	N10E6	G21E10	G23C4
1,959,540	SNV	C	T	Francci3_1630	beta-lactamase-like		Ala185Thr				99.7				
1,968,363	SNV	C	T	Francci3_1636	hypothetical protein		Ala137Val				99.7				
1,971,434	SNV	C	T	Francci3_1638	phosphoglycerate kinase		Thr285Ile	99.9		80.5					
1,981,040	SNV	C	T	Francci3_1647	glucose-6-phosphate 1-		Ala182Thr				99.5				
1,983,469	SNV	C	T									96.5			
1,983,670	SNV	C	T	Francci3_1649	protoheme IX farnesyltransferase							99.9			
1,987,548	SNV	C	T	Francci3_1653	cytochrome oxidase assembly		Gly113Ser					99.8			
1,991,103	SNV	T	C	Francci3_1657	glycerol kinase		Ser414Gly	99.8	99.8	100	99.8	99.8	99.9	100	100
1,994,633	SNV	C	T	Francci3_1660	FeS assembly protein SufB	yes			99.8						
2,009,555	SNV	G	A					99.8		82.0					
2,018,908	SNV	G	A	Francci3_1681	PilT protein-like			99.9		81.1					
2,031,399	SNV	C	T	Francci3_1693	Type IV secretory pathway VirB4 components-like		Thr228Ile					99.8			
2,032,785	SNV	C	T	Francci3_1694	Type IV secretory pathway VirD4 components-like		Thr49Ile						99.7		
2,054,984	SNV	C	T	Francci3_1721	hypothetical protein		Val45Ile						99.9		
2,059,505	SNV	G	A						99.8						
2,061,194	SNV	C	T	Francci3_1728	putative transposase		Glu398Lys						99.4		
2,068,072	SNV	C	T	Francci3_1733	hypothetical protein							93.3			
2,068,408	SNV	G	A									93.1			
2,074,164	SNV	G	A	Francci3_1739	putative ATP/GTP-binding protein		Pro215Ser					92.9			
2,076,720	SNV	C	T	Francci3_1742	hypothetical protein				99.7						
2,083,118	SNV	G	A	Francci3_1751	phosphoribosyltransferase						99.6				
2,085,324	SNV	G	A	Francci3_1753	radical SAM family protein		Thr51Ile						99.7		
2,088,365	SNV	G	A	Francci3_1756	sulfatase		Pro64Ser						99.7		
2,092,395	SNV	C	T	Francci3_1761	DNA topoisomerase II		Trp603*				99.8				
2,092,744	SNV	G	A	Francci3_1761	DNA topoisomerase II		Ala487Val						99.6		
2,096,207	Deletion	CAGCAGTT	-	Francci3_1763	DDE endonuclease		Gln56_Leu58del							NA†	
2,096,986	SNV	C	T	Francci3_1763	putative transposase						100				
2,098,180	SNV	G	A	Francci3_1764	transposase, IS4				97.6						
2,099,667	SNV	G	A	Francci3_1765	sulfatase		Gly27Glu						99.9		
2,104,142	SNV	C	T								99.8				
2,105,062	SNV	C	T	Francci3_1769	cytochrome P450								100		
2,107,470	SNV	C	T	Francci3_1771	hypothetical protein						99.8				
2,108,144	SNV	C	T	Francci3_1772	major facilitator transporter								99.6		
2,109,159	SNV	C	T	Francci3_1772	major facilitator transporter		Gly80Asp						99.4		
2,110,190	SNV	C	T	Francci3_1773	UbiE/COQ5 methyltransferase		Gly44Asp				99.6				
2,111,742	SNV	G	A	Francci3_1775	radical SAM family protein								99.7		
2,111,833	SNV	G	A	Francci3_1775	radical SAM family protein		Ser258Phe						99.9		

Table S2

Position (nt)	Mutation type*	WT allele	Mutant allele	Gene ID	Annotation	N fixation related	Amino acid change	Reads that called a mutation/all reads (%)							
								N3H4	N4H4	N6F4	N7C9	N9D9	N10E6	G21E10	G23C4
2,112,740	SNV	T	C	Francci3_1776	transposase, IS4						98.9				
2,115,581	SNV	C	T	Francci3_1778	transposase		Arg73*		99.9						
2,117,111	SNV	G	A	Francci3_1780	XRE family transcriptional regulator		Ala55Thr						99.6		
2,120,403	SNV	C	T									93.2			
2,124,905	SNV	G	A	Francci3_1787	hypothetical protein			99.6		80.1					
2,127,527	SNV	G	A	Francci3_1790	hypothetical protein								99.7		
2,129,673	SNV	C	T	Francci3_1793	phosphoesterase PHP-like		Gly183Asp						99.7		
2,130,311	SNV	C	T										99.7		
2,133,351	SNV	C	T	Francci3_1798	hypothetical protein							99.6			
2,135,659	SNV	C	T	Francci3_1801	aldo/keto reductase		Val46Met					99.7			
2,140,051	SNV	C	T	Francci3_1805	hypothetical protein		Val340Ile	99.5		80.6					
2,140,070	SNV	C	T	Francci3_1805	hypothetical protein							99.6			
2,140,329	SNV	C	T	Francci3_1805	hypothetical protein		Gly247Asp	99.4		80.2					
2,141,118	SNV	C	T	Francci3_1806	AMP-dependent synthetase and ligase		Val510Ile	99.8		83.2					
2,141,644	SNV	G	A	Francci3_1806	AMP-dependent synthetase and ligase							99.8			
2,142,637	SNV	G	A	Francci3_1806	AMP-dependent synthetase and ligase							99.4			
2,145,162	SNV	G	A	Francci3_1809	hypothetical protein								99.8		
2,146,252	SNV	G	A	Francci3_1810	uncharacterized FAD-dependent dehydrogenase		Thr280Ile						99.7		
2,148,179	SNV	G	A	Francci3_1811	NUDIX hydrolase								99.7		
2,151,726	SNV	C	T	Francci3_1816	flavoprotein		Ala100Thr				99.8				
2,151,925	SNV	C	T	Francci3_1816	flavoprotein		Trp33*				100				
2,157,603	SNV	C	T	Francci3_1821	lantibiotic dehydratase-like								99.6		
2,157,772	SNV	C	T	Francci3_1821	lantibiotic dehydratase-like		Pro449Ser						99.5		
2,158,088	SNV	C	T	Francci3_1821	lantibiotic dehydratase-like		Thr554Ile						99.8		
2,158,409	SNV	C	T	Francci3_1821	lantibiotic dehydratase-like		Ser661Phe						99.6		
2,160,900	SNV	C	T	Francci3_1823	putative O-methyltransferase		Ser6Phe						99.9		
2,162,405	SNV	C	T	Francci3_1824	hypothetical protein		Thr211Ile						99.8		
2,162,669	SNV	C	T	Francci3_1824	hypothetical protein		Ala299Val						99.4		
2,164,673	SNV	G	A	Francci3_1827	hypothetical protein								99.8		
2,165,283	SNV	G	A	Francci3_1827	hypothetical protein		Ala147Val						99.6		
2,165,920	SNV	G	A	Francci3_1828	pterin-4-alpha-carbinolamine		Ala79Val						99.8		
2,172,419	SNV	G	A									99.7			
2,172,421	SNV	G	A									99.6			
2,185,598	SNV	C	T					55.1							
2,185,650	SNV	G	A									99.6			
2,191,397	SNV	T	C										99.7		
2,196,766	SNV	C	T	Francci3_1862	aminotransferase		Ala252Val					99.8			
2,200,738	SNV	G	A	Francci3_1865	hypothetical protein		Trp10*	99.9		82.7					
2,222,006	SNV	G	A	Francci3_1889	glycoside hydrolase 15-related		Ala336Val					99.7			

Table S2

Position (nt)	Mutation type*	WT allele	Mutant allele	Gene ID	Annotation	N fixation related	Amino acid change	Reads that called a mutation/all reads (%)							
								N3H4	N4H4	N6F4	N7C9	N9D9	N10E6	G21E10	G23C4
2,222,507	SNV	G	A	Francci3_1889	glycoside hydrolase 15-related		Ala169Val					99.7			
2,222,909	SNV	T	C	Francci3_1889	glycoside hydrolase 15-related		Asp35Gly					99.9			
2,228,466	SNV	C	T	Francci3_1895	pyruvate dehydrogenase complex dehydrogenase (E1) component-like		Val508Met					99.8			
2,231,615	SNV	G	A	Francci3_1898	cation diffusion facilitator family							99.9			
2,236,684	SNV	G	A	Francci3_1903	hypothetical protein		Gly108Glu					99.9			
2,238,991	SNV	G	A	Francci3_1906	glycosyl transferase, group 1		Pro257Ser					99.8			
2,241,256	SNV	G	A									99.9			
2,241,309	SNV	G	A									99.7			
2,241,835	SNV	G	A									99.6			
2,242,307	SNV	G	A	Francci3_1909	hypothetical protein							99.9			
2,242,698	SNV	G	A	Francci3_1909	hypothetical protein		Asp282Asn					99.6			
2,242,854	SNV	G	A	Francci3_1910	urate oxidase								99.8		
2,243,560	SNV	G	A	Francci3_1910	urate oxidase		Ala45Val						100		
2,244,799	SNV	G	A	Francci3_1911	ErfK/YbiS/YcfS/YnhG		Ala125Thr					99.7			
2,245,123	SNV	C	T	Francci3_1911	ErfK/YbiS/YcfS/YnhG		Pro233Ser				99.6				
2,246,290	SNV	G	A	Francci3_1912	hypothetical protein		Val16Ile					99.6			
2,247,357	SNV	G	A									100			
2,248,135	SNV	G	A	Francci3_1913	OmpA/MotB								99.7		
2,249,777	SNV	G	A	Francci3_1915	hypothetical protein							99.9			
2,251,330	SNV	G	A	Francci3_1917	GntR family transcriptional regulator		Pro217Leu					94.8			
2,251,686	SNV	G	A	Francci3_1917	GntR family transcriptional regulator							99.4			
2,255,617	SNV	C	T	Francci3_1919	LysR family transcriptional regulator		Ala26Val						99.8		
2,258,306	SNV	C	T	Francci3_1922	transposase IS66		Ala484Thr						98.6		
2,258,584	SNV	G	A	Francci3_1922	transposase IS66		Ala391Val					97.5			
2,260,519	SNV	A	G	Francci3_1923	FAD dependent oxidoreductase		Ser531Pro						99.9		
2,261,396	SNV	C	T	Francci3_1923	FAD dependent oxidoreductase								99.9		
2,261,574	SNV	G	A	Francci3_1923	FAD dependent oxidoreductase		Ala179Val					99.8			
2,262,193	SNV	G	A									99.7			
2,262,325	SNV	C	T									99.7			
2,262,814	SNV	G	A	Francci3_1924	hypothetical protein		Val135Met					99.5			
2,262,939	SNV	G	A	Francci3_1924	hypothetical protein							99.8			
2,263,129	SNV	C	T	Francci3_1924	hypothetical protein		Leu240Phe					99.4			
2,266,613	SNV	C	T	Francci3_1929	AMP-dependent synthetase and ligase		Gly575Glu					99.6			
2,268,156	SNV	G	A	Francci3_1929	AMP-dependent synthetase and ligase		Pro61Ser					99.7			
2,268,296	SNV	G	A	Francci3_1929	AMP-dependent synthetase and ligase		Ala14Val					99.4			
2,269,076	SNV	G	A	Francci3_1930	cytochrome P450		Ala12Thr					99.5			
2,269,696	SNV	C	T	Francci3_1930	cytochrome P450							89.9			
2,282,841	SNV	G	A	Francci3_1942	uptake hydrogenase large subunit (Hydrogenlyase) (hupL1), FRAAL2393	yes	Arg448His				99.7				

Table S2

Position (nt)	Mutation type*	WT allele	Mutant allele	Gene ID	Annotation	N fixation related	Amino acid change	Reads that called a mutation/all reads (%)							
								N3H4	N4H4	N6F4	N7C9	N9D9	N10E6	G21E10	G23C4
2,290,151	SNV	C	T									99.8			
2,290,652	SNV	C	T	Francci3_1949	cold-shock DNA-binding protein family protein		Pro20Leu					99.8			
2,304,604	SNV	G	A	Francci3_1962	transposase, IS4			99.3		58.4					
2,329,076	SNV	G	A	Francci3_1983	D-lactate dehydrogenase			99.8		84.6					
2,330,578	SNV	G	A	Francci3_1983	D-lactate dehydrogenase		Trp789*						99.8		
2,337,453	SNV	C	T	Francci3_1989	putative PAS/PAC sensor protein		Gly252Asp	99.5		79.4					
2,340,301	SNV	G	A	Francci3_1990	metallophosphoesterase		Pro96Ser					99.8			
2,344,405	SNV	C	T	Francci3_1994	phytanoyl-CoA dioxygenase								99.7		
2,344,488	SNV	C	T	Francci3_1995	hypothetical protein								99.6		
2,351,531	SNV	C	T	Francci3_2001	homoserine O-succinyltransferase		Asp51Asn						99.8		
2,354,935	SNV	C	T										99.8		
2,355,723	SNV	C	T	Francci3_2006	antibiotic biosynthesis		Glu100Lys						99.8		
2,357,873	SNV	C	T	Francci3_2007	FAD linked oxidase-like		Pro511Ser						99.8		
2,357,916	SNV	C	T	Francci3_2007	FAD linked oxidase-like		Pro525Leu						99.8		
2,359,193	SNV	C	T	Francci3_2007	FAD linked oxidase-like		Pro951Ser						99.8		
2,361,585	SNV	G	A	Francci3_2009	cytochrome-c oxidase								100		
2,362,144	SNV	G	A										99.8		
2,362,685	SNV	G	A	Francci3_2011	hypothetical protein		Arg6Gln						99.8		
2,366,992	Deletion	GCGACCGCCCGGGAC	-	Francci3_2015	MarR family transcriptional regulator		Thr23_Ala27 del	50.5	56.4	57.9	54.7	54.4	54.9		
2,403,991	SNV	C	T	Francci3_2043	2-polyprenyl-6-methoxyphenol hydroxylase and related FAD-dependent oxidoreductases-like		Ala463Thr				99.8				
2,419,643	SNV	C	T	Francci3_2055	haloacid dehalogenase-like hydrolase		Ala167Val					99.7			
2,447,331	SNV	C	T						99.1						
2,447,877	SNV	A	G	Francci3_2080	transposase, IS4				100						
2,456,458	SNV	C	T	Francci3_2088	recombinase		Ala463Val	99.4		80.8					
2,457,794	SNV	C	T	Francci3_2089	hypothetical protein		Gly3Glu					99.5			
2,462,707	SNV	C	T	Francci3_2095	putative 3-oxoacyl-ACP synthase III			50.3							
2,484,710	SNV	C	T	Francci3_2118	oligopeptide/dipeptide ABC transporter, ATP-binding protein-like		Ala131Val	99.6		82.8					
2,484,730	SNV	C	T	Francci3_2118	oligopeptide/dipeptide ABC transporter, ATP-binding protein-like		Arg138Trp				99.4				
2,485,067	SNV	G	A	Francci3_2118	oligopeptide/dipeptide ABC transporter, ATP-binding protein-like		Gly250Asp					99.3			
2,485,624	SNV	C	T	Francci3_2118	oligopeptide/dipeptide ABC transporter, ATP-binding protein-like		Pro436Ser	99.8		79.8					

Table S2

Position (nt)	Mutation type*	WT allele	Mutant allele	Gene ID	Annotation	N fixation related	Amino acid change	Reads that called a mutation/all reads (%)							
								N3H4	N4H4	N6F4	N7C9	N9D9	N10E6	G21E10	G23C4
2,489,356	SNV	G	A						99.8						
2,489,470	SNV	G	A								99.5				
2,509,415	SNV	G	A	Francci3_2140	hypothetical protein		Val216Ile						99.8		
2,511,085	SNV	G	A									99.9			
2,515,210	SNV	G	A	Francci3_2147	phage integrase								100		
2,515,738	SNV	C	T	Francci3_2148	hypothetical protein		Pro102Ser				99.3				
2,518,868	SNV	G	A	Francci3_2150	IS630 family transposase		Ala85Val						99.9		
2,522,063	SNV	G	A	Francci3_2153	response regulator receiver protein		Ala161Val					99.6			
2,523,025	SNV	G	A	Francci3_2154	periplasmic sensor signal transduction histidine kinase		Ser412Phe						100		
2,523,735	SNV	G	A	Francci3_2154	periplasmic sensor signal transduction histidine kinase							99.7			
2,525,546	SNV	G	A	Francci3_2156	phosphonate ABC transporter ATP-				99.9						
2,528,383	SNV	G	A										99.9		
2,529,681	SNV	G	A	Francci3_2160	hypothetical protein								99.8		
2,529,854	SNV	G	A	Francci3_2160	hypothetical protein		Ser70Asn						99.7		
2,531,506	SNV	G	A									99.5			
2,531,592	SNV	G	A										99.9		
2,531,805	SNV	G	A										99.8		
2,533,010	SNV	G	A	Francci3_2163	short-chain dehydrogenase/reductase		Ala156Thr						99.6		
2,533,852	SNV	C	T	Francci3_2164	LacI family transcription regulator		Gly257Asp		99.8						
2,534,154	SNV	G	A	Francci3_2164	LacI family transcription regulator								99.9		
2,537,454	SNV	G	A	Francci3_2166	citrate lyase		Val122Met						100		
2,544,814	SNV	G	A	Francci3_2172	GrpE protein		Ala117Val						99.8		
2,546,271	SNV	G	A	Francci3_2173	molecular chaperone DnaK		Ala281Val						99.9		
2,548,147	SNV	G	A	Francci3_2174	heat shock protein Hsp20		Gly160Asp						99.6		
2,555,545	SNV	C	T	Francci3_2181	cobyrinic acid a,c-diamide synthase						99.6				
2,555,992	SNV	C	T	Francci3_2181	cobyrinic acid a,c-diamide synthase						99.6				
2,567,471	SNV	G	A	Francci3_2196	hypothetical protein							99.9			
2,568,407	SNV	C	T	Francci3_2196	hypothetical protein						99.8				
2,568,550	SNV	C	T								99.4				
2,569,339	SNV	C	T	Francci3_2197	RNA-directed DNA polymerase		Pro33Leu		99.9						
2,569,614	SNV	C	T	Francci3_2197	RNA-directed DNA polymerase		Arg125Cys					99.3			
2,570,025	SNV	G	A	Francci3_2197	RNA-directed DNA polymerase		Ala262Thr						99.7		
2,570,559	SNV	C	T	Francci3_2197	RNA-directed DNA polymerase		Pro440Ser						99.7		
2,570,919	SNV	G	A	Francci3_2198	hypothetical protein		Ala45Thr					99.8			
2,572,419	SNV	C	T	Francci3_2199	epoxide hydrolase-like		Ala10Thr					99.7			
2,574,164	SNV	G	A	Francci3_2202	hypothetical protein		Trp32*					99.8			

Table S2

Position (nt)	Mutation type*	WT allele	Mutant allele	Gene ID	Annotation	N fixation related	Amino acid change	Reads that called a mutation/all reads (%)							
								N3H4	N4H4	N6F4	N7C9	N9D9	N10E6	G21E10	G23C4
2,574,401	SNV	G	A	Francci3_2202	hypothetical protein							99.4			
2,590,719	Insertion	-	GGGG TAGC						71.6		72.9	71.9	70.0	NA†	NA†
2,593,217	SNV	G	A	Francci3_2218	hypothetical protein		Val22Ile				99.6				
2,596,567	SNV	G	A	Francci3_2222	HNH endonuclease							99.9			
2,600,163	SNV	G	A	Francci3_2226	GCN5-related N-acetyltransferase		Thr12Met				99.9				
2,630,292	SNV	G	A	Francci3_2252	extracellular solute-binding protein			99.7		77.9					
2,637,427	SNV	G	A	Francci3_2258	two component LuxR family transcriptional regulator							99.7			
2,644,346	SNV	C	T	Francci3_2264	resolvase-like protein							99.5			
2,647,733	SNV	C	T	Francci3_2270	hypothetical protein								99.0		
2,649,580	SNV	G	A	Francci3_2272	apolipoprotein N-acyltransferase		Ala381Val	99.9		86.8					
2,650,276	SNV	G	A	Francci3_2272	apolipoprotein N-acyltransferase		Pro149Leu	99.6		79.6					
2,650,791	SNV	C	T										98.4		
2,658,088	SNV	A	G	Francci3_2277	zinc-binding alcohol dehydrogenase		Thr379Ala						100		
2,658,886	SNV	C	T	Francci3_2278	hypothetical protein								99.8		
2,659,711	SNV	C	T	Francci3_2278	hypothetical protein							99.6			
2,665,131	SNV	G	A	Francci3_2285	hypothetical protein								99.6		
2,666,307	SNV	G	A										99.8		
2,666,319	SNV	G	A										99.5		
2,670,596	SNV	G	A	Francci3_2289	major facilitator transporter		Pro223Ser						99.7		
2,670,761	SNV	C	T	Francci3_2289	major facilitator transporter		Ala168Thr						99.6		
2,670,904	SNV	C	T	Francci3_2289	major facilitator transporter		Gly120Asp						99.7		
2,672,757	SNV	G	A					50.7							
2,674,349	SNV	G	A	Francci3_2294	monooxygenase, FAD-binding								99.8		
2,674,693	SNV	C	T	Francci3_2295	lignostilbene-alpha beta-dioxygenase and related enzymes-like		Gly550Glu						99.8		
2,682,691	SNV	G	A	Francci3_2300	pentapeptide repeat-containing protein								99.7		
2,687,044	SNV	C	T									99.6			
2,694,931	SNV	G	A										99.8		
2,726,353	SNV	G	A	Francci3_2352	hypothetical protein		Ala503Val						99.8		
2,735,510	SNV	G	A	Francci3_2361	hypothetical protein		Ala577Val					99.0			
2,741,567	SNV	C	T	Francci3_2367	transposase Tn3				99.9						
2,745,115	SNV	G	A								99.6				
2,763,949	SNV	C	T	Francci3_2388	hypothetical protein		Arg354Gln	52.5							
2,780,773	SNV	C	T	Francci3_2401	two component transcriptional		Gly111Glu		77.8						
2,814,916	SNV	G	A	Francci3_2423	extracellular solute-binding protein						99.6				
2,820,312	SNV	G	A	Francci3_2427	cysteine synthase		Pro95Ser						99.8		
2,828,659	SNV	C	T											100	
2,847,828	SNV	C	T	Francci3_2450	amino acid adenylation		Ala3716Thr					83.8			

Table S2

Position (nt)	Mutation type*	WT allele	Mutant allele	Gene ID	Annotation	N fixation related	Amino acid change	Reads that called a mutation/all reads (%)							
								N3H4	N4H4	N6F4	N7C9	N9D9	N10E6	G21E10	G23C4
2,855,357	SNV	T	C	Francci3 2450	amino acid adenylation		Glu1206Gly						80.0		
2,855,359	SNV	G	C	Francci3 2450	amino acid adenylation								80.0		
2,886,853	SNV	C	T	Francci3 2460	amino acid adenylation		Val434Ile						99.9		
2,892,325	SNV	C	T	Francci3 2461	amino acid adenylation		Trp3215*	99.3		84.4					
2,895,966	SNV	A	C	Francci3 2461	amino acid adenylation		Trp2002Gly			47.9					
2,898,345	SNV	C	T	Francci3 2461	amino acid adenylation		Ala1209Thr	97.2		81.6					
2,902,343	SNV	C	T	Francci3 2462	ABC transporter related			99.7		83.0					
2,902,897	SNV	G	A	Francci3 2462	ABC transporter related		Leu346Phe					87.9			
2,914,160	SNV	G	A	Francci3 2468	carbon starvation protein CstA		Ala201Val	99.9		83.3					
2,915,638	Deletion	CTGG AAT	-								99.1	99.6			
2,916,240	SNV	G	A	Francci3 2471	Na+/solute symporter							99.9			
2,925,962	SNV	T	C	Francci3 2481	transposase, IS4				98.5		99.3	95.3	97.3		
2,928,108	SNV	G	A						99.8						
2,936,221	SNV	G	A						99.5						
2,947,154	SNV	T	C	Francci3 2496	radical SAM family protein		Leu301Pro		99.8						
2,960,274	SNV	G	A	Francci3 2507	5-oxoprolinase (ATP-hydrolyzing)		Thr569Ile						99.9		
2,974,730	SNV	G	A	Francci3 2518	methane/phenol/toluene hydroxylase				99.8						
2,976,478	SNV	T	C	Francci3 2519	oxidoreductase FAD-binding region								100		
2,980,116	SNV	G	A	Francci3 2522	transposase IS66				96.6						
2,982,200	SNV	C	T					99.9		81.6					
2,985,499	SNV	G	A										99.8		
2,988,114	SNV	G	A					99.7		81.7					
2,989,076	SNV	C	T	Francci3 2527	NAD-dependent		Arg107Cys				99.5				
2,992,228	SNV	G	A	Francci3 2529	recombinase		Pro12Ser						99.7		
2,998,098	SNV	G	A	Francci3 2534	transposase, IS4								99.3		
3,001,372	SNV	G	A	Francci3 2537	alpha/beta hydrolase fold								99.8		
3,007,272	SNV	G	A	Francci3 2544	hypothetical protein		Asp170Asn						99.4		
3,007,508	SNV	G	A										99.7		
3,008,143	SNV	G	A	Francci3 2545	PfkB		Leu106Phe						99.8		
3,008,193	SNV	G	A	Francci3 2545	PfkB		Thr89Ile						99.6		
3,010,769	SNV	G	A										99.7		
3,010,817	SNV	G	A										99.9		
3,013,452	SNV	C	T	Francci3 2550	hypothetical protein		Ser76Phe					99.7			
3,014,419	SNV	G	A										99.7		
3,019,475	Deletion	ATGG CGGG ACGC	-									61.6			
3,021,492	SNV	C	T	Francci3 2560	hypothetical protein		Ala43Val						99.7		
3,031,508	SNV	G	A	Francci3 2572	hypothetical protein						99.3				

Table S2

Position (nt)	Mutation type*	WT allele	Mutant allele	Gene ID	Annotation	N fixation related	Amino acid change	Reads that called a mutation/all reads (%)							
								N3H4	N4H4	N6F4	N7C9	N9D9	N10E6	G21E10	G23C4
3,038,290	SNV	C	T	Francci3_2575	hypothetical protein								99.9		
3,052,873	SNV	C	T	Francci3_2589	hypothetical protein								99.6		
3,055,005	SNV	C	T	Francci3_2590	putative phosphoesterase		Ala432Thr						99.8		
3,057,112	SNV	C	T	Francci3_2591	Dyp-type peroxidase		Val319Ile						99.8		
3,057,641	SNV	C	T	Francci3_2591	Dyp-type peroxidase								100		
3,058,077	SNV	C	T										99.9		
3,058,273	SNV	C	T										99.8		
3,059,768	SNV	C	T	Francci3_2592	monooxygenase, FAD-binding								99.9		
3,060,926	SNV	C	T	Francci3_2594	TetR family transcriptional regulator			99.9		83.7					
3,062,894	SNV	C	T									87.7			
3,069,424	SNV	G	A						99.7						
3,071,689	SNV	C	T	Francci3_2602	ABC transporter related									100	99.3
3,079,415	SNV	G	A	Francci3_2608	hypothetical protein		Arg69Cys		99.9						
3,084,354	SNV	G	A	Francci3_2613	hypothetical protein								99.8		
3,085,288	SNV	G	A									89.4			
3,107,651	SNV	C	T	Francci3_2633	peptidase M50		Gly208Asp					99.7			
3,107,811	SNV	C	T	Francci3_2633	peptidase M50		Ala155Thr					99.8			
3,110,428	SNV	C	T	Francci3_2635	ABC transporter related		Ala270Val					99.6			
3,112,350	SNV	C	T	Francci3_2636	hypothetical protein								99.5		
3,118,047	SNV	C	T	Francci3_2642	cysteinyl-tRNA synthetase							99.8			
3,120,848	SNV	C	T	Francci3_2645	phosphoglycerate mutase		Ala112Thr					99.7			
3,133,202	SNV	C	T	Francci3_2653	hypothetical protein		Gly12Glu					86.2			
3,133,364	SNV	G	A									99.8			
3,136,220	SNV	G	A	Francci3_2656	2-methylcitrate dehydratase							98.9			
3,136,480	SNV	C	T	Francci3_2656	2-methylcitrate dehydratase		Gly316Ser		99.6						
3,138,785	SNV	G	A	Francci3_2658	FAD linked oxidase-like		Leu400Phe				99.3				
3,143,724	SNV	C	T	Francci3_2663	urease accessory protein UreG							87.4			
3,146,271	SNV	G	A	Francci3_2666	MMPL		Pro774Ser				99.4				
3,146,377	SNV	G	A	Francci3_2666	MMPL						99.5				
3,147,367	SNV	G	A	Francci3_2666	MMPL						99.6				
3,148,088	SNV	G	A	Francci3_2666	MMPL		Ala168Val				99.6				
3,153,716	SNV	C	T								99.9				
3,154,094	SNV	C	T	Francci3_2671	pyridoxamine 5'-phosphate oxidase-related, FMN-binding		Thr94Ile						99.9		
3,154,356	SNV	C	T	Francci3_2672	hypothetical protein		Gly295Asp				99.7				
3,161,056	SNV	C	T	Francci3_2675	hypothetical protein		Val27Ile				99.6				
3,161,554	SNV	C	T	Francci3_2676	helicase-like							99.8			
3,161,597	SNV	C	T	Francci3_2676	helicase-like		Arg1221His				99.6				
3,164,451	SNV	C	T	Francci3_2676	helicase-like		Glu270Lys				99.9				
3,164,779	SNV	C	T	Francci3_2676	helicase-like		Met160Ile				99.8				

Table S2

Position (nt)	Mutation type*	WT allele	Mutant allele	Gene ID	Annotation	N fixation related	Amino acid change	Reads that called a mutation/all reads (%)							
								N3H4	N4H4	N6F4	N7C9	N9D9	N10E6	G21E10	G23C4
3,165,207	SNV	G	A	Francci3_2676	helicase-like		Pro18Ser						99.2		
3,165,233	SNV	C	T	Francci3_2676	helicase-like		Gly9Glu				99.3				
3,172,028	SNV	C	T	Francci3_2686	Rieske (2Fe-2S) protein						99.6				
3,172,864	SNV	C	T	Francci3_2687	Rieske (2Fe-2S) protein						99.4				
3,173,445	SNV	C	T	Francci3_2687	Rieske (2Fe-2S) protein		Ala215Val				99.8				
3,178,963	SNV	C	T	Francci3_2691	hypothetical protein		Gln294*				99.8				
3,179,495	SNV	C	T								99.8				
3,185,820	SNV	C	T	Francci3_2700	putative signal transduction histidine kinase		Ser96Asn				99.7				
3,186,186	SNV	C	T	Francci3_2701	hypothetical protein		Asp88Asn					99.7			
3,186,943	SNV	T	C	Francci3_2702	XRE family transcriptional regulator		Leu117Pro					89.5			
3,189,527	SNV	C	T	Francci3_2705	arginyl-tRNA synthetase		Ala558Thr				99.3				
3,196,317	SNV	C	T	Francci3_2711	pyridoxal-dependent decarboxylase		Ala310Thr		99.8						
3,197,712	SNV	C	T	Francci3_2712	transposase, IS4				83.3						
3,210,187	SNV	C	T	Francci3_2725	hypothetical protein						99.7				
3,235,920	SNV	C	T										99.6		
3,242,134	SNV	G	A	Francci3_2751	putative transcriptional regulator		Pro84Ser					99.7			
3,248,325	SNV	C	T	Francci3_2756	tryptophan halogenase		Pro492Ser		99.6						
3,254,741	SNV	C	T	Francci3_2761	luciferase-like		Val99Met					99.6			
3,255,851	SNV	C	T	Francci3_2762	putative methyltransferase		Ala64Thr					99.6			
3,257,037	SNV	G	A	Francci3_2763	methyltransferase type 11		Gly189Glu				99.5				
3,273,394	SNV	C	T	Francci3_2778	hypothetical protein						99.3				
3,286,894	Deletion	CGAG GATG GTTA	-					89.5		74.0					
3,293,005	SNV	G	A	Francci3_2796	ATPase, E1-E2 type		Ala504Val						99.8		
3,293,165	SNV	G	A	Francci3_2796	ATPase, E1-E2 type		Leu451Phe						99.8		
3,294,594	SNV	G	A										99.7		
3,295,501	SNV	G	A	Francci3_2797	undecaprenyl pyrophosphate								99.9		
3,296,914	SNV	G	A	Francci3_2799	transposase, IS4		Thr339Ile						94.4		
3,298,340	SNV	G	A	Francci3_2800	hypothetical protein								99.8		
3,299,617	SNV	C	T	Francci3_2801	hypothetical protein		Gly60Asp						99.8		
3,303,740	SNV	C	T	Francci3_2804	citrate lyase								99.8		
3,306,974	SNV	G	A	Francci3_2808	hypothetical protein		Ala207Val						99.6		
3,308,146	SNV	C	T	Francci3_2809	hypothetical protein		Arg67His						99.9		
3,310,180	SNV	C	T	Francci3_2811	serine/threonine protein kinase		Arg26Trp							100	
3,311,477	SNV	C	T	Francci3_2811	serine/threonine protein kinase		Ala458Val					100			
3,311,532	SNV	C	T	Francci3_2811	serine/threonine protein kinase								99.9		
3,312,292	SNV	C	T	Francci3_2811	serine/threonine protein kinase		Leu730Phe						99.8		
3,313,599	SNV	C	T	Francci3_2813	extracellular solute-binding protein		Met78Ile						99.8		

Table S2

Position (nt)	Mutation type*	WT allele	Mutant allele	Gene ID	Annotation	N fixation related	Amino acid change	Reads that called a mutation/all reads (%)							
								N3H4	N4H4	N6F4	N7C9	N9D9	N10E6	G21E10	G23C4
3,315,505	SNV	G	A	Francci3_2814	hypothetical protein							99.9			
3,316,503	SNV	G	A	Francci3_2816	hypothetical protein							99.8			
3,316,595	SNV	C	T	Francci3_2816	hypothetical protein		Gly168Arg						99.9		
3,317,759	SNV	C	T										99.6		
3,318,989	SNV	C	T	Francci3_2818	bacterioferritin		Trp135*					99.9			
3,320,429	SNV	C	T	Francci3_2819	hypothetical protein		Gly14Asp					99.2			
3,321,176	SNV	C	T										99.3		
3,323,977	SNV	C	T	Francci3_2823	L-proline dehydrogenase		Asp135Asn						99.9		
3,324,558	SNV	C	T	Francci3_2824	hypothetical protein								99.5		
3,324,806	SNV	C	T	Francci3_2824	hypothetical protein		Ala23Thr						99.6		
3,324,939	SNV	G	A									100			
3,326,242	SNV	G	A	Francci3_2826	ATP-binding region, ATPase-like								99.9		
3,326,346	SNV	C	T	Francci3_2826	ATP-binding region, ATPase-like		Ala706Thr						99.5		
3,328,211	SNV	C	T	Francci3_2826	ATP-binding region, ATPase-like		Gly84Asp						99.9		
3,328,631	SNV	C	T										99.7		
3,331,991	SNV	C	T	Francci3_2829	HNH endonuclease		Ala46Thr					99.7			
3,332,318	SNV	C	T									99.9			
3,333,914	SNV	C	T	Francci3_2830	O-acetylhomoserine aminocarboxypropyltransferase							99.5			
3,335,029	SNV	G	A	Francci3_2831	homoserine O-acetyltransferase		Ala265Thr						99.8		
3,335,859	SNV	G	A	Francci3_2832	RpiR family transcriptional regulator		Ala95Thr						99.6		
3,336,478	SNV	G	A	Francci3_2832	RpiR family transcriptional regulator		Gly301Asp						98.2		
3,338,787	SNV	G	A	Francci3_2834	extracellular solute-binding protein		Asp232Asn						99.8		
3,340,697	SNV	G	A	Francci3_2836	metallophosphoesterase		Gly88Asp					99.4			
3,341,056	SNV	G	A	Francci3_2836	metallophosphoesterase		Ala208Thr					99.5			
3,341,598	SNV	C	T	Francci3_2837	binding-protein-dependent transport systems inner membrane component		Pro66Leu						99.7		
3,342,853	SNV	C	T	Francci3_2838	binding-protein-dependent transport systems inner membrane component								100		
3,343,119	SNV	G	A	Francci3_2838	binding-protein-dependent transport systems inner membrane component							99.9			
3,343,758	SNV	C	T	Francci3_2839	McrBC 5-methylcytosine restriction system component-like								99.7		
3,344,089	SNV	C	T	Francci3_2839	McrBC 5-methylcytosine restriction system component-like		Gly203Asp						99.9		
3,345,737	SNV	C	T	Francci3_2840	AAA_5 ATPase associated with various cellular activities		Gly397Glu						99.5		
3,346,037	SNV	G	A	Francci3_2840	AAA_5 ATPase associated with various cellular activities		Ala297Val					99.6			

Table S2

Position (nt)	Mutation type*	WT allele	Mutant allele	Gene ID	Annotation	N fixation related	Amino acid change	Reads that called a mutation/all reads (%)							
								N3H4	N4H4	N6F4	N7C9	N9D9	N10E6	G21E10	G23C4
3,346,126	SNV	G	A	Francci3_2840	AAA_5 ATPase associated with various cellular activities							99.7			
3,346,642	SNV	G	A	Francci3_2840	AAA_5 ATPase associated with various cellular activities							99.7			
3,346,719	SNV	C	T	Francci3_2840	AAA_5 ATPase associated with various cellular activities		Ala70Thr						99.5		
3,347,869	SNV	C	T	Francci3_2841	zeta toxin		Pro171Ser					99.7			
3,349,258	SNV	C	T	Francci3_2842	DEAD/DEAH box helicase-like							86.9			
3,349,947	SNV	C	T									99.6			
3,353,517	SNV	C	T	Francci3_2845	DNA helicase, putative								99.8		
3,357,930	SNV	C	T	Francci3_2846	hypothetical protein		Gly235Asp						99.7		
3,359,267	SNV	C	T	Francci3_2847	antibiotic biosynthesis								99.7		
3,360,272	SNV	C	T	Francci3_2849	glucose 1-dehydrogenase		Gly132Arg						100		
3,362,428	SNV	G	A	Francci3_2851	beta-ketoacyl synthase		Pro349Ser						99.8		
3,364,703	SNV	G	A	Francci3_2854	phosphopantetheine-binding		Gln17*						99.9		
3,366,314	SNV	G	A	Francci3_2856	regulatory protein, LuxR		Ala193Val						99.9		
3,368,971	SNV	G	A	Francci3_2860	periplasmic sensor signal transduction histidine kinase		Pro855Leu						99.8		
3,371,640	SNV	G	A										99.9		
3,372,258	SNV	G	A										99.5		
3,373,619	SNV	G	A	Francci3_2862	hypothetical protein		Val56Met						99.6		
3,375,650	SNV	G	A	Francci3_2864	hypothetical protein		Ala132Thr						99.7		
3,378,917	SNV	G	A										99.7		
3,393,009	SNV	C	T	Francci3_2875	serine/threonine protein kinase		Thr485Ile						99.7		
3,407,566	SNV	C	T	Francci3_2893	hypothetical protein			99.5		79.7					
3,413,886	SNV	C	T								99.8				
3,417,611	SNV	G	A	Francci3_2902	hypothetical protein		Gln239*		100						
3,418,389	SNV	C	T										99.5		
3,427,787	SNV	C	T	Francci3_2910	asparagine synthase, glutamine-hydrolyzing		Gly92Ser		99.6						
3,429,300	SNV	G	A	Francci3_2911	hypothetical protein							88.7			
3,429,358	SNV	G	A	Francci3_2911	hypothetical protein		Ala521Val					90.3			
3,434,839	SNV	G	A	Francci3_2915	hypothetical protein		Ala370Val	99.3		79.4					
3,443,564	SNV	C	T					99.8		80.3					
3,453,287	SNV	C	T	Francci3_2930	Beta-hydroxyacyl dehydratase		Gly553Glu						99.5		
3,453,299	SNV	C	T	Francci3_2930	Beta-hydroxyacyl dehydratase		Arg549Gln						99.8		
3,459,061	SNV	C	T	Francci3_2931	beta-ketoacyl synthase		Val1203Met						99.7		
3,459,518	SNV	C	T	Francci3_2931	beta-ketoacyl synthase								100		
3,478,720	SNV	C	T	Francci3_2944	aldehyde dehydrogenase		Gln348*						99.8		
3,478,739	SNV	C	T	Francci3_2944	aldehyde dehydrogenase		Ser354Phe		99.4						

Table S2

Position (nt)	Mutation type*	WT allele	Mutant allele	Gene ID	Annotation	N fixation related	Amino acid change	Reads that called a mutation/all reads (%)							
								N3H4	N4H4	N6F4	N7C9	N9D9	N10E6	G21E10	G23C4
3,479,504	SNV	G	A	Francci3_2945	zinc-binding alcohol dehydrogenase		Val74Met		99.8						
3,484,871	SNV	C	T	Francci3_2949	catalase		Gly419Asp				99.8				
3,488,104	SNV	C	T	Francci3_2950	CoA-binding								99.8		
3,499,816	SNV	G	A	Francci3_2954	bacteriophage resistance gene PglY		Glu726Lys					99.8			
3,507,925	SNV	G	A	Francci3_2958	transcriptional modulator of MazE/toxin, MazF							99.9			
3,517,804	SNV	G	A	Francci3_2974	allophanate hydrolase subunit 1		Gly82Ser				99.5				
3,532,065	SNV	C	T	Francci3_2982	acyl-CoA synthetase		Leu173Phe					99.8			
3,540,845	Deletion	CGGC AACC	-								73.9	75.3			
3,563,162	SNV	G	A												100
3,576,431	SNV	G	A	Francci3_3013	glutamate synthase (NADH) large								99.4		
3,589,696	SNV	C	T	Francci3_3023	phosphoribosyl isomerase A		Ala37Thr	99.7		80.1					
3,589,960	SNV	C	T	Francci3_3024	imidazole glycerol phosphate synthase subunit HisH		Asp196Asn		99.9						
3,590,050	SNV	C	T	Francci3_3024	imidazole glycerol phosphate synthase subunit HisH		Asp166Asn		99.8						
3,634,715	SNV	C	T	Francci3_3065	stress protein		Val32Met						85.5		
3,638,260	SNV	C	T	Francci3_3070	thiamine-phosphate								99.8		
3,638,769	SNV	C	T	Francci3_3070	thiamine-phosphate		Gly65Arg						99.6		
3,639,747	SNV	G	A	Francci3_3073	oxidoreductase-like		Ala540Val					99.8			
3,651,220	SNV	G	A	Francci3_3081	XRE family transcriptional regulator				99.9						
3,651,410	SNV	C	T	Francci3_3082	hypothetical protein		Glu245Lys						99.7		
3,651,646	SNV	G	A	Francci3_3082	hypothetical protein		Ala166Val				99.5				
3,666,124	SNV	C	T	Francci3_3094	arsenite-transporting ATPase		Gly169Asp					99.6			
3,673,048	SNV	G	A										99.8		
3,678,218	SNV	G	A	Francci3_3105	cytochrome b/b6-like								99.8		
3,678,330	SNV	G	A	Francci3_3105	cytochrome b/b6-like		Thr332Ile						99.8		
3,681,125	SNV	C	T	Francci3_3107	cytochrome c, class I		Ala145Thr				99.9				
3,682,437	SNV	C	T								99.4				
3,716,754	SNV	C	T									99.7			
3,724,379	SNV	G	A	Francci3_3146	NH(3)-dependent NAD(+) synthetase		Asp478Asn			74.6					
3,724,698	SNV	C	T	Francci3_3146	NH(3)-dependent NAD(+) synthetase		Thr584Ile	99.4		77.5					
3,734,368	SNV	G	A	Francci3_3156	NUDIX hydrolase							99.6			
3,737,009	SNV	G	A	Francci3_3158	DNA repair protein RecN		Ala582Val					99.0			
3,739,077	SNV	G	A	Francci3_3159	inorganic polyphosphate/ATP-NAD		Pro285Ser					99.9			
3,741,550	SNV	G	A	Francci3_3162	hypothetical protein		Arg29His						99.4		
3,745,620	SNV	G	A								99.7				
3,768,414	SNV	C	T	Francci3_3178	PAS/PAC sensor signal transduction histidine kinase		Cys96Tyr							100	

Table S2

Position (nt)	Mutation type*	WT allele	Mutant allele	Gene ID	Annotation	N fixation related	Amino acid change	Reads that called a mutation/all reads (%)							
								N3H4	N4H4	N6F4	N7C9	N9D9	N10E6	G21E10	G23C4
3,770,913	SNV	G	A	Francci3_3182	translation initiation factor IF-3				76.2						
3,787,684	SNV	G	A	Francci3_3196	putative integration host factor MihF								99.8		
3,789,942	SNV	G	A	Francci3_3198	carbamoyl phosphate synthase large subunit		Ala736Val					99.8			
3,792,393	SNV	C	T	Francci3_3199	carbamoyl phosphate synthase small subunit								100		
3,811,975	SNV	C	T					99.8		81.5					
3,813,189	SNV	G	C	Francci3_3218	GntR family transcriptional regulator		Asp240His			52.6					
3,814,964	SNV	G	A	Francci3_3219	glutamyl-tRNA synthetase										100
3,819,167	SNV	C	T	Francci3_3226	hypothetical protein		Ala270Thr		77.8						
3,828,109	SNV	C	T	Francci3_3233	methyltransferase type 11		Arg214Trp					99.5			
3,828,526	SNV	G	A	Francci3_3234	MMPL		Gly45Glu		99.8						
3,847,879	SNV	G	A	Francci3_3249	serine phosphatase		Arg29Cys				99.9				
3,852,650	SNV	C	T	Francci3_3253	two component LuxR family transcriptional regulator		Gln21*					88.5			
3,855,625	SNV	C	T	Francci3_3256	WD-40 repeat-containing serine/threonin protein kinase		Gly758Asp	99.8		84.8					
3,860,379	SNV	C	T	Francci3_3258	acyltransferase 3								99.7		
3,861,842	SNV	C	T	Francci3_3258	acyltransferase 3		Ala504Val						99.4		
3,863,591	SNV	G	A	Francci3_3259	glycoside hydrolase family protein		Glu313Lys	99.9		82.6					
3,865,429	SNV	G	A								99.9				
3,867,264	SNV	G	A						99.8						
3,876,184	SNV	G	A	Francci3_3273	methionyl-tRNA synthetase		Ala30Val				99.6				
3,905,461	SNV	C	T					99.4		76.8					
3,907,940	SNV	C	T	Francci3_3300	hypothetical protein							99.9			
3,908,721	SNV	C	T					98.6		80.6					
3,913,948	SNV	G	A	Francci3_3304	hypothetical protein		Pro103Leu		100						
3,915,655	SNV	C	T	Francci3_3305	Type IV secretory pathway VirD4 components-like		Ala238Thr	99.8		81.4					
3,916,878	SNV	C	T	Francci3_3306	hypothetical protein		Ala126Thr	100		84.6					
3,917,772	SNV	C	T	Francci3_3307	zinc finger, CHC2-type		Ala33Val	99.4		80.1					
3,921,569	SNV	C	T					99.9		81.7					
3,924,365	SNV	C	T					99.5		80.0					
3,925,842	SNV	C	T					98.3		81.9					
3,931,147	SNV	C	T	Francci3_3316	protein-L-isoaspartate(D-aspartate) O-methyltransferase			99.9		79.7					
3,931,346	SNV	G	A	Francci3_3316	protein-L-isoaspartate(D-aspartate) O-methyltransferase		Ala158Val				99.8				
3,931,993	SNV	C	T	Francci3_3317	hypothetical protein			99.7		83.6					
3,935,706	SNV	G	A								99.6				

Table S2

Position (nt)	Mutation type*	WT allele	Mutant allele	Gene ID	Annotation	N fixation related	Amino acid change	Reads that called a mutation/all reads (%)							
								N3H4	N4H4	N6F4	N7C9	N9D9	N10E6	G21E10	G23C4
3,939,194	SNV	C	T	Francci3_3323	hypothetical protein		Gly45Asp				99.3				
3,952,049	SNV	G	A	Francci3_3334	hypothetical protein								99.8		
3,969,971	SNV	G	A	Francci3_3346	CRISPR-associated Cas1 family		Val69Ile				99.6				
3,977,872	SNV	G	A									99.8			
3,978,174	SNV	G	A									99.9			
3,979,030	SNV	G	A	Francci3_3355	benzoate membrane transport protein							99.3			
3,996,291	SNV	G	A	Francci3_3367	hypothetical protein		Pro154Leu	99.6		75.1					
3,996,872	SNV	C	T	Francci3_3368	aminoglycoside phosphotransferase		Gly229Asp				99.9				
3,997,837	SNV	C	T	Francci3_3369	aminotransferase		Gly350Ser					99.7			
4,008,937	SNV	C	T	Francci3_3378	tRNA-guanine transglycosylases, various specificities		Pro118Ser				99.4				
4,018,637	SNV	C	T	Francci3_3390	phage integrase			99.7		81.8					
4,020,009	SNV	A	G	Francci3_3391	hypothetical protein			99.6		84.9					
4,026,294	SNV	G	A	Francci3_3395	WD-40 repeat-containing serine/threonin protein kinase								99.6		
4,034,756	Deletion	CTCCGGACCACCGGAC	-								60.9	64.8			
4,035,282	SNV	C	T	Francci3_3403	iron permease FTR1		Ala48Val					99.7			
4,039,342	SNV	C	T	Francci3_3405	twin-arginine translocation pathway							99.9			
4,040,944	SNV	C	T	Francci3_3406	AAA ATPase, central region		His105Tyr					100.0			
4,041,649	SNV	C	T	Francci3_3406	AAA ATPase, central region		Leu340Phe				99.8				
4,042,831	SNV	G	A	Francci3_3407	hypothetical protein		Ala199Val					99.7			
4,050,559	SNV	C	T	Francci3_3410	serine/threonine protein kinase							99.8			
4,050,596	SNV	G	A	Francci3_3410	serine/threonine protein kinase		Thr359Ile				99.8				
4,062,530	SNV	G	A	Francci3_3418	sporulation and cell division protein		Thr56Ile				99.9				
4,077,762	SNV	G	A										99.9		
4,078,191	SNV	G	A	Francci3_3428	limonene-1,2-epoxide hydrolase						99.9				
4,105,091	SNV	C	T	Francci3_3448	ATP-dependent helicase HrpA			99.9		78.2					
4,112,048	SNV	G	A								99.9				
4,112,150	SNV	G	A	Francci3_3452	galactose-1-phosphate						99.8				
4,115,757	SNV	G	A	Francci3_3455	putative lipoprotein				99.9						
4,115,971	SNV	G	A	Francci3_3455	putative lipoprotein		Val279Ile						99.8		
4,116,060	SNV	C	T	Francci3_3455	putative lipoprotein			99.7		81.6					
4,117,252	SNV	G	A	Francci3_3456	ABC transporter related			99.9		75.3					
4,122,751	SNV	G	A	Francci3_3458	hypothetical protein		Asp207Asn				99.7				
4,124,763	SNV	G	A	Francci3_3459	LmbE-like protein		Ser195Phe				99.5				
4,131,455	SNV	C	T	Francci3_3466	succinate dehydrogenase flavoprotein subunit		Thr152Ile				99.7				

Table S2

Position (nt)	Mutation type*	WT allele	Mutant allele	Gene ID	Annotation	N fixation related	Amino acid change	Reads that called a mutation/all reads (%)							
								N3H4	N4H4	N6F4	N7C9	N9D9	N10E6	G21E10	G23C4
4,134,555	SNV	G	A	Francci3_3468	hypothetical protein		Val82Ile						99.5		
4,141,278	SNV	T	C	Francci3_3473	polysaccharide biosynthesis protein						99.7				
4,142,231	SNV	C	T	Francci3_3473	polysaccharide biosynthesis protein		Ala330Val						99.8		
4,146,774	SNV	G	A	Francci3_3477	protein tyrosine/serine phosphatase		Arg126His				99.7				
4,151,690	SNV	C	T	Francci3_3482	3-oxoacyl-(acyl carrier protein)		Ala181Val					99.8			
4,163,311	SNV	G	A					99.6		77.9					
4,163,452	SNV	G	A	Francci3_3492	polyphosphate glucokinase		Asp22Asn	99.8		76.3					
4,164,803	SNV	C	T	Francci3_3493	hypothetical protein		Ala105Val					99.9			
4,174,807	SNV	C	T									99.9			
4,179,430	SNV	C	T	Francci3_3502	GCN5-related N-acetyltransferase						99.7				
4,193,449	SNV	G	A									99.9			
4,204,252	SNV	G	A	Francci3_3519	ABC transporter related		Ala154Thr				99.7				
4,210,502	SNV	G	A	Francci3_3525	recombinase A		Ala258Val						99.7		
4,211,711	SNV	C	T								99.7				
4,212,938	SNV	C	T	Francci3_3527	DEAD/DEAH box helicase-like						99.8				
4,225,523	SNV	C	T	Francci3_3536	Ferritin and Dps		Gly80Asp				99.7				
4,227,241	SNV	C	T	Francci3_3538	competence/damage-inducible protein cinA		Ala177Thr					99.8			
4,238,625	SNV	G	A	Francci3_3546	putative phosphoketolase		His788Tyr					99.9			
4,239,283	SNV	G	A	Francci3_3546	putative phosphoketolase							99.9			
4,239,785	SNV	C	T	Francci3_3546	putative phosphoketolase		Gly401Glu					99.5			
4,241,259	SNV	C	T	Francci3_3547	FAD-dependent thymidylate synthase							99.7			
4,242,637	SNV	C	T	Francci3_3548	histidyl-tRNA synthetase							99.5			
4,247,822	SNV	C	T	Francci3_3551	NAD-dependent		Trp133*					99.8			
4,275,934	SNV	C	T	Francci3_3576	Poly(3-hydroxybutyrate)		Ala65Thr					99.8			
4,280,143	SNV	G	A								99.7				
4,280,300	SNV	C	T	Francci3_3581	30S ribosomal protein S2		Ala271Thr					99.8			
4,280,511	SNV	G	A	Francci3_3581	30S ribosomal protein S2						99.8				
4,283,443	SNV	G	A	Francci3_3583	phage integrase						99.6				
4,289,431	SNV	G	A	Francci3_3589	signal peptidase I		Ser51Phe					99.7			
4,291,483	SNV	G	A	Francci3_3592	16S rRNA-processing protein RimM								99.6		
4,300,269	SNV	C	T										99.7		
4,309,087	SNV	G	A	Francci3_3607	DEAD/DEAH box helicase-like								99.6		
4,311,699	SNV	G	A	Francci3_3610	hypothetical protein								99.8		
4,316,493	SNV	C	T	Francci3_3616	hypothetical protein				99.2						
4,316,565	SNV	C	T	Francci3_3616	hypothetical protein			99.5		83.9					
4,318,604	SNV	G	A	Francci3_3617	diguanylate		Pro321Ser						99.9		
4,318,719	SNV	C	T	Francci3_3617	diguanylate				99.7						
4,320,396	SNV	C	T	Francci3_3618	polyphosphate kinase		Pro190Leu		99.8						
4,321,740	SNV	G	A	Francci3_3618	polyphosphate kinase		Gly638Asp						99.7		

Table S2

Position (nt)	Mutation type*	WT allele	Mutant allele	Gene ID	Annotation	N fixation related	Amino acid change	Reads that called a mutation/all reads (%)							
								N3H4	N4H4	N6F4	N7C9	N9D9	N10E6	G21E10	G23C4
4,324,078	SNV	G	A	Francci3_3621	isopropylmalate isomerase large		Pro299Ser	99.7		80.6					
4,334,155	SNV	C	T	Francci3_3628	putative alpha-isopropylmalate/homocitrate synthase		Asp22Asn		99.9						
4,334,210	SNV	C	T	Francci3_3628	putative alpha-isopropylmalate/homocitrate synthase			99.5		80.8					
4,336,481	SNV	A	G	Francci3_3631	branched chain amino acid aminotransferase apoenzyme		Phe74Ser							100	
4,338,422	SNV	C	T										99.7		
4,348,522	SNV	C	T	Francci3_3640	acetolactate synthase 1 catalytic							99.7			
4,355,050	SNV	G	A	Francci3_3646	amino acid-binding ACT		Met5Ile		99.8		99.5	99.9	99.8	100	100
4,355,057	SNV	C	T	Francci3_3646	amino acid-binding ACT								99.9		
4,355,723	SNV	C	T	Francci3_3646	amino acid-binding ACT								99.8		
4,355,917	SNV	C	T	Francci3_3647	DNA ligase, NAD-dependent								99.7		
4,356,316	SNV	C	T	Francci3_3647	DNA ligase, NAD-dependent								99.6		
4,358,537	SNV	C	T	Francci3_3648	methionine synthase, vitamin-B12 independent		Val160Ile						99.8		
4,361,052	SNV	C	T	Francci3_3650	aminotransferase, class V		Val148Met						99.7		
4,362,552	SNV	G	A	Francci3_3652	methylmalonyl-CoA mutase								99.7		
4,373,891	SNV	C	T	Francci3_3654	hypothetical protein		Pro67Ser						99.9		
4,379,737	SNV	C	T	Francci3_3659	electron transfer flavoprotein beta-		Val26Ile					99.9			
4,382,126	SNV	G	A	Francci3_3661	endonuclease/exonuclease/phosphata						100				
4,383,758	SNV	G	A	Francci3_3662	short chain enoyl-CoA hydratase		Ala24Val					99.4			
4,398,993	SNV	G	A	Francci3_3671	hypothetical protein		Pro272Leu	99.9		81.7					
4,399,066	SNV	C	T	Francci3_3671	hypothetical protein		Ala248Thr						99.9		
4,399,717	SNV	C	T	Francci3_3671	hypothetical protein		Gly31Ser				99.9				
4,404,561	SNV	C	T	Francci3_3673	heat shock protein 70		Ala436Thr						99.9		
4,409,865	SNV	C	T	Francci3_3677	stress protein		Val160Ile				99.0				
4,411,335	SNV	G	A	Francci3_3678	alpha amylase, catalytic region						99.6				
4,425,098	SNV	C	T	Francci3_3688	hypothetical protein		Ala114Thr				99.5				
4,425,145	SNV	C	T	Francci3_3688	hypothetical protein		Gly98Asp				99.7				
4,427,600	SNV	T	C	Francci3_3691	hypothetical protein			99.7		82.3					
4,435,001	SNV	C	T	Francci3_3700	UDP-N-acetylglucosamine 1-carboxyvinyltransferase		Gly359Asp		99.9						
4,439,700	SNV	G	A	Francci3_3706	F0F1 ATP synthase subunit epsilon				99.8						
4,453,074	SNV	G	A	Francci3_3720	peptide chain release factor 1		Leu206Phe	99.7		79.8					
4,473,194	SNV	G	A	Francci3_3737	hypothetical protein		Ser135Phe						99.9		
4,476,180	SNV	C	T	Francci3_3740	diguanylate							99.7			
4,476,740	SNV	C	T	Francci3_3740	diguanylate		Pro433Leu				99.7				
4,482,367	SNV	G	A	Francci3_3744	UvrD/REP helicase		Ala910Val	99.7		79.2					
4,482,379	SNV	G	A	Francci3_3744	UvrD/REP helicase		Ala906Val	100		78.3					

Table S2

Position (nt)	Mutation type*	WT allele	Mutant allele	Gene ID	Annotation	N fixation related	Amino acid change	Reads that called a mutation/all reads (%)							
								N3H4	N4H4	N6F4	N7C9	N9D9	N10E6	G21E10	G23C4
4,497,809	SNV	G	A	Francci3 3749	hypothetical protein		Asp56Asn					84.0			
4,509,799	SNV	C	T	Francci3 3760	signal transduction histidine kinase		Asp31Asn		99.8						
4,514,801	SNV	C	T	Francci3 3764	putative PAS/PAC sensor protein						99.1				
4,519,473	SNV	G	A								99.4	99.6			
4,562,405	SNV	G	A	Francci3 3805	DNA end-binding protein Ku					79.1					
4,568,687	SNV	G	A									84.9			
4,575,284	SNV	G	A	Francci3 3817	MgtE intracellular region						99.9				
4,590,662	SNV	G	A	Francci3 3829	sec-independent translocase		Pro137Leu		99.4						
4,591,556	SNV	G	A	Francci3 3830	peptidase S1 and S6,				99.9						
4,597,252	SNV	C	T	Francci3 3835	hypothetical protein		Ala241Val						99.7		
4,606,090	SNV	G	A	Francci3 3845	4Fe-4S ferredoxin, iron-sulfur binding			99.6		80.1					
4,608,629	SNV	G	A	Francci3 3847	hypothetical protein		Pro450Ser		99.1						
4,615,290	SNV	C	T	Francci3 3852	glycosyl transferase family protein		Ala271Thr						99.7		
4,615,935	SNV	C	T	Francci3 3852	glycosyl transferase family protein		Asp56Asn						99.7		
4,616,110	SNV	T	C					99.3	99.3	99.8	98.8	99.3	99.4	100	100
4,624,021	SNV	G	A	Francci3 3860	hypothetical protein		Ala252Thr		99.8						
4,635,179	SNV	G	A	Francci3 3872	hypothetical protein		Val140Ile					100			
4,635,430	SNV	C	T	Francci3 3872	hypothetical protein								99.8		
4,642,688	SNV	C	T	Francci3 3880	exodeoxyribonuclease VII large								99.6		
4,643,281	SNV	C	T	Francci3 3880	exodeoxyribonuclease VII large		Ala31Thr						99.8		
4,645,458	SNV	G	A	Francci3 3882	fumarate hydratase		Gly231Asp				99.9				
4,653,127	SNV	C	T	Francci3 3889	HAD family hydrolase		Val225Met						99.8		
4,654,249	SNV	C	T										99.5		
4,655,001	SNV	C	T	Francci3 3891	hypothetical protein								99.6		
4,655,459	SNV	C	T	Francci3 3892	hypothetical protein								99.5		
4,655,533	SNV	C	T	Francci3 3892	hypothetical protein		Val336Met						99.7		
4,655,641	SNV	C	T	Francci3 3892	hypothetical protein		Ala300Thr						99.7		
4,656,097	SNV	G	A	Francci3 3892	hypothetical protein		Pro148Ser	99.9		84.4					
4,657,453	SNV	C	T	Francci3 3894	hypothetical protein		Pro48Ser						99.8		
4,659,481	SNV	C	T	Francci3 3896	PhoH-like protein		Ala131Thr						99.5		
4,660,058	SNV	C	T										99.7		
4,660,969	SNV	C	T										99.7		
4,661,017	SNV	C	T										99.7		
4,663,667	SNV	C	T	Francci3 3898	hemolysin III family channel protein		Pro225Ser						100		
4,663,860	SNV	C	T	Francci3 3898	hemolysin III family channel protein		Thr289Ile						99.5		
4,664,071	SNV	C	T	Francci3 3899	hypothetical protein		Ser145Asn						99.9		
4,665,432	SNV	C	T	Francci3 3900	hypothetical protein								99.9		
4,669,042	SNV	C	T	Francci3 3901	leucyl-tRNA synthetase								99.8		
4,670,458	SNV	C	T	Francci3 3902	hypothetical protein		Met60Ile				99.8				
4,670,697	SNV	C	T										99.6		

Table S2

Position (nt)	Mutation type*	WT allele	Mutant allele	Gene ID	Annotation	N fixation related	Amino acid change	Reads that called a mutation/all reads (%)							
								N3H4	N4H4	N6F4	N7C9	N9D9	N10E6	G21E10	G23C4
4,671,570	SNV	C	T	Francci3_3903	LmbE-like protein		Gly51Arg						99.6		
4,676,197	SNV	C	T	Francci3_3906; Francci3_3907	von Willebrand factor type A; PadR family transcriptional regulator		Ala644Val; Gly203Asp						99.4		
4,703,838	SNV	C	T					99.8		80.3					
4,703,915	SNV	C	T					99.9		80.3					
4,711,315	SNV	C	T	Francci3_3936	lipopolysaccharide biosynthesis		Val438Ile					99.8			
4,711,554	SNV	G	A	Francci3_3936	lipopolysaccharide biosynthesis		Thr358Ile					99.6			
4,711,872	SNV	C	T	Francci3_3936	lipopolysaccharide biosynthesis		Trp252*						99.8		
4,715,947	SNV	C	T	Francci3_3938	methyltransferase type 12									100	
4,719,423	SNV	C	T	Francci3_3942	serine O-acetyltransferase		Gly204Asp					99.4			
4,737,977	SNV	C	T	Francci3_3959	ABC transporter related		Leu447Phe		99.8						
4,745,038	SNV	G	A								99.7				
4,754,472	SNV	C	T	Francci3_3973	FHA domain-containing protein		Ala79Val				100				
4,758,440	SNV	C	T	Francci3_3976	glycoside hydrolase family protein		Leu82Phe				99.9				
4,773,593	SNV	C	T	Francci3_3989	recombinase		Ala384Thr					99.7			
4,794,428	SNV	C	T	Francci3_4011	HsdR family type I site-specific deoxyribonuclease		Ala735Thr						99.5		
4,794,495	SNV	G	A	Francci3_4011	HsdR family type I site-specific deoxyribonuclease						99.6				
4,799,573	SNV	C	T	Francci3_4013	transposase, IS4						95.0				
4,801,533	SNV	G	A	Francci3_4014	N-6 DNA methylase							99.8			
4,806,111	SNV	G	A	Francci3_4019	hypothetical protein					80.4					
4,813,327	SNV	G	A	Francci3_4026	AMP-dependent synthetase and ligase		Val326Ile				99.7				
4,832,251	SNV	C	T	Francci3_4047	WD-40 repeat-containing serine/threonin protein kinase				99.7						
4,836,174	SNV	G	A	Francci3_4049	GAF sensor hybrid histidine kinase		Ala922Val			77.6					
4,856,100	SNV	C	T	Francci3_4059	L-glutamine synthetase		Pro441Leu				99.8				
4,859,393	SNV	A	G	Francci3_4061	TetR family transcriptional regulator		*196Trp	99.7	99.8	100	99.7	99.9	99.8	100	100
4,860,306	SNV	C	T	Francci3_4062	hypothetical protein		Ala144Thr						99.8		
4,864,209	SNV	G	A	Francci3_4064	methyltransferase FkbM		Pro88Ser				99.8	99.5			
4,865,296	SNV	C	T	Francci3_4065	glycine dehydrogenase		Gly905Ser				99.9				
4,875,183	SNV	G	A					99.3							
4,878,114	SNV	C	T						99.8						
4,887,526	SNV	G	A	Francci3_4081	putative transposase		Ser200Phe				99.8				
4,888,665	SNV	G	A	Francci3_4082	DNA polymerase III subunit alpha		Arg88His				97.8				
4,893,058	SNV	G	A					87.4		75.5					
4,902,305	SNV	G	A	Francci3_4093	NUDIX hydrolase		Glu159Lys					99.7			
4,913,282	SNV	G	A	Francci3_4105	zinc-binding alcohol dehydrogenase						99.6				
4,913,293	SNV	G	A	Francci3_4105	zinc-binding alcohol dehydrogenase		Gly292Asp				99.8				
4,915,823	SNV	G	A	Francci3_4110	integrase								99.3		

Table S2

Position (nt)	Mutation type*	WT allele	Mutant allele	Gene ID	Annotation	N fixation related	Amino acid change	Reads that called a mutation/all reads (%)							
								N3H4	N4H4	N6F4	N7C9	N9D9	N10E6	G21E10	G23C4
4,917,086	SNV	G	A	Francci3_4112	IS630 family transposase		Ala133Val				99.3				
4,918,142	SNV	C	T										99.6		
4,923,071	SNV	G	A	Francci3_4120	transposase, IS4						99.7				
4,923,851	SNV	G	A	Francci3_4120	transposase, IS4								99.8		
4,926,075	SNV	G	A										99.9		
4,926,615	SNV	G	A	Francci3_4124	recombinase		Gly170Asp					99.9			
4,931,383	SNV	G	A	Francci3_4129	hypothetical protein								99.8		
4,932,472	SNV	C	T	Francci3_4131	hypothetical protein		Val162Met				99.4				
4,932,863	SNV	C	T	Francci3_4131	hypothetical protein						99.3				
4,935,872	SNV	C	T	Francci3_4134	NDP-hexose 2,3-dehydratase		Gly307Asp						99.9		
4,937,206	SNV	C	T	Francci3_4135	methyltransferase type 11		Arg41Trp					99.8			
4,938,840	SNV	C	T	Francci3_4137	TetR family transcriptional regulator		Ala110Val						99.6		
4,940,338	SNV	C	T	Francci3_4138	short chain dehydrogenase		Ala294Val						99.4		
4,942,380	SNV	G	A	Francci3_4140	luciferase-like		Thr75Ile						98.8		
4,942,522	SNV	G	A	Francci3_4140	luciferase-like								99.7		
4,944,666	SNV	G	A	Francci3_4143	actinorhodin polyketide synthase bifunctional cyclase/dehydratase								99.7		
4,948,178	SNV	G	A	Francci3_4147	phosphopantetheine-binding								100		
4,948,658	SNV	G	A	Francci3_4148	beta-ketoacyl synthase								99.6		
4,948,776	SNV	G	A	Francci3_4148	beta-ketoacyl synthase		Thr144Ile						99.6		
4,950,736	SNV	C	T	Francci3_4151	actinorhodin polyketide synthase bifunctional cyclase/dehydratase								99.9		
4,955,100	SNV	G	A	Francci3_4154	EmrB/QacA family drug resistance transporter							99.8			
4,958,784	SNV	T	C	Francci3_4157	hypothetical protein		Val99Ala				99.9				
4,960,995	SNV	G	A	Francci3_4159	hypothetical protein		Pro239Leu				99.6				
4,962,125	SNV	G	A	Francci3_4160	hypothetical protein		Val127Met						99.3		
4,963,998	SNV	G	A	Francci3_4162	putative replication initiation protein						98.9				
4,969,175	SNV	G	A	Francci3_4167	putative transposase		Ala182Thr				99.5				
4,972,732	SNV	C	T	Francci3_4168	DNA polymerase III subunit alpha		Gly67Arg	99.7		83.3					
4,972,956	SNV	C	T										99.2		
4,974,021	SNV	C	T	Francci3_4170	hypothetical protein		Ala204Thr						99.3		
4,974,358	SNV	G	A	Francci3_4170	hypothetical protein						99.8				
4,976,568	SNV	C	T										99.8		
4,977,825	SNV	G	A	Francci3_4174	copper resistance protein CopC		Ala211Val				99.7				
4,977,937	SNV	G	A	Francci3_4174	copper resistance protein CopC		Leu174Phe				99.5				
4,978,451	SNV	G	A	Francci3_4174	copper resistance protein CopC						99.7				
4,980,130	SNV	C	T										99.5		
4,981,889	SNV	C	T	Francci3_4178	monooxygenase, FAD-binding		Thr120Ile						99.7		
4,982,951	SNV	C	T	Francci3_4178	monooxygenase, FAD-binding		Ala474Val						99.5		

Table S2

Position (nt)	Mutation type*	WT allele	Mutant allele	Gene ID	Annotation	N fixation related	Amino acid change	Reads that called a mutation/all reads (%)							
								N3H4	N4H4	N6F4	N7C9	N9D9	N10E6	G21E10	G23C4
4,983,018	SNV	C	T	Francci3 4178	monooxygenase, FAD-binding								99.6		
4,983,593	SNV	C	T	Francci3 4179	hypothetical protein								99.8		
4,984,371	SNV	C	T	Francci3 4180	PAS/PAC sensor hybrid histidine		Gly475Asp						99.2		
4,984,742	SNV	C	T	Francci3 4180	PAS/PAC sensor hybrid histidine								99.5		
4,986,672	SNV	C	T	Francci3 4182	hypothetical protein								90.6		
4,990,321	SNV	G	A	Francci3 4184	hypothetical protein		Val628Ile						99.9		
4,995,924	SNV	G	A	Francci3 4186	tetratricopeptide TPR 4								99.7		
4,997,001	SNV	G	A	Francci3 4186	tetratricopeptide TPR 4								99.7		
4,997,188	SNV	G	A	Francci3 4186	tetratricopeptide TPR 4		Ala436Val						99.9		
4,997,315	SNV	G	A	Francci3 4186	tetratricopeptide TPR 4		Pro394Ser						99.9		
4,998,685	SNV	C	T	Francci3 4187	biotin synthase		Asp317Asn						99.6		
5,005,062	SNV	G	A	Francci3 4194	methyltransferase type 12							86.8			
5,008,835	SNV	G	A	Francci3 4196	hypothetical protein		Glu656Lys				99.7				
5,018,351	SNV	C	T	Francci3 4203	3-(3-hydroxyphenyl)propionate		Glu255Lys				99.6				
5,024,447	SNV	C	T										99.4		
5,032,002	SNV	C	T	Francci3 4214	insertion element hypothetical protein		Pro411Ser						99.4		
5,040,629	SNV	G	A										99.9		
5,040,683	SNV	G	A	Francci3 4225	putative transposase		Ser69Phe						99.9		
5,042,811	SNV	C	T	Francci3 4227	transposase, IS4		Gly119Glu					99.5			
5,044,962	SNV	C	T	Francci3 4229	hypothetical protein		Gly76Glu					99.8			
5,050,489	SNV	G	A	Francci3 4232	nitroreductase		Gln16*				99.7				
5,054,194	SNV	C	T	Francci3 4235	peptidase S15				100		99.9	99.9	99.8	100	100
5,068,192	SNV	G	A										99.8		
5,068,708	SNV	G	A	Francci3 4246	RNA methyltransferase TrmH, group 3								99.8		
5,069,109	SNV	G	A	Francci3 4246	RNA methyltransferase TrmH, group 3		Arg61Cys						99.4		
5,069,824	SNV	G	A	Francci3 4247	hypothetical protein		Gly98Asp						99.7		
5,071,828	SNV	G	A	Francci3 4249	hypothetical protein		Val116Ile			81.1					
5,071,851	SNV	G	A	Francci3 4249	hypothetical protein								99.9		
5,073,324	SNV	G	A	Francci3 4250	FHA domain-containing protein								99.8		
5,075,444	SNV	G	A	Francci3_4252	pyridoxamine 5'-phosphate oxidase-related, FMN-binding								99.9		
5,079,030	SNV	C	T	Francci3_4257	hypothetical protein									100	100
5,090,842	SNV	G	A	Francci3 4267	prolyl-tRNA synthetase		Ala270Thr					99.8			
5,097,056	SNV	G	A						99.7						
5,098,314	SNV	G	A										99.5		
5,100,908	SNV	C	T					99.8		80.6					
5,109,124	SNV	C	T	Francci3 4278	transcription factor WhiB								99.6		
5,111,402	SNV	G	A	Francci3 4280	anion-transporting ATPase		Pro124Leu					88.5			
5,119,246	SNV	C	T	Francci3 4286	Crp/FNR family transcriptional		Asp30Asn		78.2						
5,119,629	SNV	G	A	Francci3 4287	hypothetical protein		Ala31Val	99.9		79.7					

Table S2

Position (nt)	Mutation type*	WT allele	Mutant allele	Gene ID	Annotation	N fixation related	Amino acid change	Reads that called a mutation/all reads (%)							
								N3H4	N4H4	N6F4	N7C9	N9D9	N10E6	G21E10	G23C4
5,121,291	Deletion	CCCACCGGCACCGTCC	-	Francci3_4290	NUDIX hydrolase		Ala265_Pro270del		59.5		55.2	57.1	56.1		
5,144,752	SNV	G	A	Francci3_4306	thymidylate kinase							99.8			
5,145,924	SNV	G	A	Francci3_4307	DNA polymerase III subunit delta'		Gly352Asp					99.8			
5,146,450	SNV	G	A	Francci3_4308	PSP1		Val62Ile					99.9			
5,148,596	SNV	G	A	Francci3_4310	inorganic diphosphatase							99.8			
5,151,658	SNV	G	A	Francci3_4312	hypothetical protein							99.6			
5,154,998	SNV	C	T	Francci3_4314	membrane protease FtsH catalytic		Ala210Val							100	100
5,161,777	SNV	G	A	Francci3_4320	hypothetical protein		Asp120Asn				100				
5,173,858	SNV	C	T	Francci3_4331	hypothetical protein							99.7			
5,189,244	SNV	C	T	Francci3_4347	glycoside hydrolase family protein		Ala635Thr					99.7			
5,190,651	SNV	C	T	Francci3_4347	glycoside hydrolase family protein		Val166Ile					99.8			
5,193,966	SNV	G	A	Francci3_4350	protein of unknown function DUF224, cysteine-rich region		Pro343Ser					99.7			
5,199,143	SNV	G	A	Francci3_4353	GrpE protein						99.9				
5,200,133	SNV	T	C	Francci3_4354	chaperone DnaJ-like				99.8						
5,200,362	SNV	C	T	Francci3_4354	chaperone DnaJ-like		Arg144Cys				99.6				
5,202,857	SNV	C	T	Francci3_4356	ATPase AAA-2		Arg333Cys				99.7				
5,203,730	SNV	C	T	Francci3_4356	ATPase AAA-2		Leu624Phe				99.9				
5,204,177	SNV	G	A	Francci3_4356	ATPase AAA-2		Asp773Asn					99.7			
5,204,478	SNV	C	T								99.8				
5,205,587	SNV	G	A	Francci3_4358	membrane-flanked domain-containing protein							99.8			
5,205,770	SNV	C	T	Francci3_4358	membrane-flanked domain-containing protein						99.9				
5,207,371	SNV	G	A	Francci3_4359	hypothetical protein		Pro54Ser					99.5			
5,208,833	SNV	G	A	Francci3_4361	NAD-dependent		Pro342Ser						99.6		
5,219,718	SNV	G	A	Francci3_4370	phosphoribosylamine--glycine ligase							99.7			
5,261,001	SNV	G	A	Francci3_4400	helicase-like		Ala77Val					99.7			
5,261,202	SNV	G	A	Francci3_4400	helicase-like		Ser10Leu					99.9			
5,268,561	SNV	C	T	Francci3_4407	dihydropteroate synthase		Gly132Asp	99.9		85.1					
5,268,997	SNV	G	A	Francci3_4408	GCN5-related N-acetyltransferase			99.7		77.7					
5,312,875	SNV	G	A	Francci3_4445	hypothetical protein		Gly76Asp				99.7				
5,340,439	SNV	G	A	Francci3_4468	hypothetical protein		Ala10Thr	99.6		82.9					
5,358,845	SNV	G	A	Francci3_4487	nifD; nitrogenase molybdenum-iron protein alpha chain	yes	Thr285Ile				99.7				
5,358,859	SNV	G	A	Francci3_4487	nifD; nitrogenase molybdenum-iron protein alpha chain	yes					99.8				

Table S2

Position (nt)	Mutation type*	WT allele	Mutant allele	Gene ID	Annotation	N fixation related	Amino acid change	Reads that called a mutation/all reads (%)							
								N3H4	N4H4	N6F4	N7C9	N9D9	N10E6	G21E10	G23C4
5,360,263	SNV	C	T	Francci3_4488	nifH; nitrogenase iron protein subunit	yes	Glu115Lys				99.7				
5,360,424	SNV	G	A	Francci3_4488	nifH; nitrogenase iron protein subunit	yes	Ala61Val				99.9				
5,368,010	SNV	G	A	Francci3_4495; Francci3_4496	peptidase M52; nickel-dependent hydrogenase, large subunit		; Pro4Ser				99.7				
5,378,755	SNV	G	A										99.6		
5,387,103	SNV	G	A	Francci3_4512	hypothetical protein		Trp302*						99.5		
5,389,116	SNV	C	T	Francci3_4514	hypothetical protein		Ser215Phe						99.9		
5,389,227	SNV	T	C	Francci3_4514	hypothetical protein		Val252Ala				99.8				
5,389,470	SNV	C	T	Francci3_4514	hypothetical protein		Ala333Val						99.7		
5,391,574	SNV	C	T										99.6		
5,393,406	SNV	C	T	Francci3_4518	primary replicative DNA helicase								99.9		
5,399,712	SNV	C	T	Francci3_4525	hypothetical protein		Arg336His		99.9		99.8	99.6	99.9	100	100
5,401,127	SNV	C	T	Francci3_4526	glycosyl transferase family protein		Gly946Asp				99.6				
5,408,700	SNV	C	T	Francci3_4531	metal dependent phosphohydrolase		Val493Ile		99.8		99.6	99.9	99.6	100	100
5,416,789	SNV	C	T	Francci3_4534	serine/threonine protein kinase		Ala127Val						99.9		
5,417,385	SNV	G	A	Francci3_4534	serine/threonine protein kinase		Ala326Thr				99.9				
5,417,403	SNV	G	A	Francci3_4534	serine/threonine protein kinase		Ala332Thr				99.8				
5,421,853	SNV	G	A	Francci3_4537	thioredoxin		Met74Ile				99.7				
5,425,394	SNV	G	A	Francci3_4540	chromosome segregation DNA-binding protein						99.5				
5,427,163	SNV	G	A	Francci3_4542	16S rRNA methyltransferase GidB			99.8		76.2					
5,427,709	Insertion	-	C					54.2							
5,430,367	SNV	C	T	Francci3_4545	60 kDa inner membrane insertion		Gly445Asp				99.5				

*SNV represents single nucleotide variation.

†Mutation was detected but the value was not assigned.
